# Probing the Photochemical Formation of Hydroxyl Radical from Dissolved Organic Matter: Insights into the H_2_O_2_-Dependent Pathway

**DOI:** 10.1021/acs.est.4c10348

**Published:** 2025-01-17

**Authors:** Kai Cheng, Hang Li, Juliana R. Laszakovits, Charles M. Sharpless, Fernando Rosario-Ortiz, Garrett McKay

**Affiliations:** † Zachry Department of Civil & Environmental Engineering, Texas A&M University, College Station, Texas 77843, United States; ‡ Department of Environmental Systems Science, ETH Zurich, 8092 Zurich, Switzerland; § Andlinger Center for Energy and the Environment, Princeton University, Princeton, New Jersey 08540, United States; ∥ Department of Civil, Environmental and Architectural Engineering, University of Colorado, Boulder, Colorado 80309, United States; ⊥ Environmental Engineering Program, University of Colorado Boulder, Boulder, Colorado 80309, United States

**Keywords:** dissolved organic matter, hydroxyl radical, hydrogen peroxide, iron, one-electron reductant, ketyl radical

## Abstract

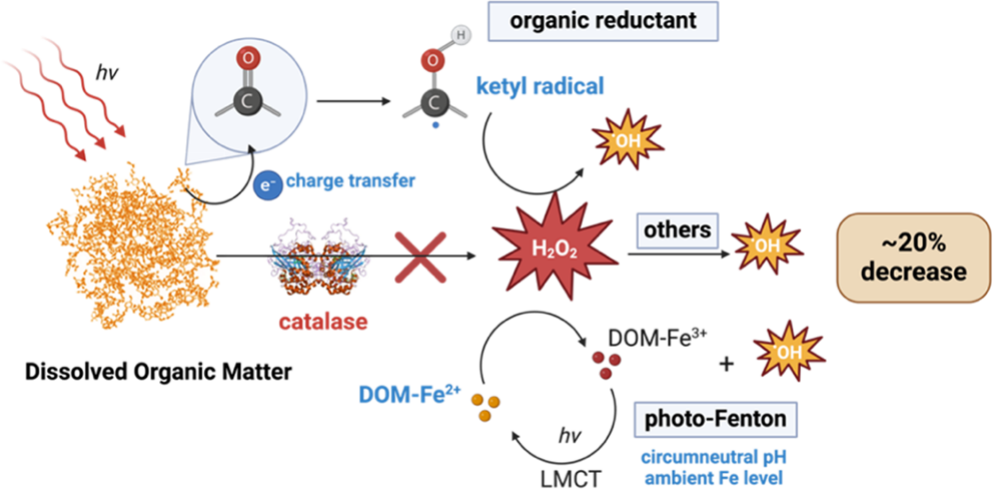

This study quantifies the contribution of the H_2_O_2_-dependent pathway to hydroxyl radical (^•^OH) production from the photolysis of dissolved organic matter (DOM). ^•^OH formation rates were cross-validated using benzoate and terephthalate as probe compounds for diverse DOM sources (reference isolates and whole waters). Catalase addition revealed that the H_2_O_2_-dependent pathway accounts for 10–20% of the total ^•^OH production in DOM isolate materials, but no significant correlation was observed between ambient iron (Fe) concentrations and H_2_O_2_-dependent ^•^OH formation. This lack of correlation was likely due to lower total Fe levels in isolated materials, thus limiting the concentration of photochemically produced Fe(II) available for reaction with H_2_O_2_. Notably, the H_2_O_2_-dependent pathway contributed 11 ± 3% to ^•^OH formation from Pony Lake fulvic acid, which had the lowest Fe content, implicating additional H_2_O_2_-driven formation mechanisms independent of Fe. Experiments with the DOM model compounds acetophenone and *p*-benzoquinone indicated no ^•^OH production from triplet DOM reactions with H_2_O_2_. However, ^•^OH formation rate increased 6-fold when H_2_O_2_ was reduced by ketyl radicals formed from the reaction between excited triplet acetophenone and 2,4,6-trimethylphenol. This study advances the knowledge of ^•^OH production mechanisms from DOM photolysis, providing insight into the role of H_2_O_2_ in aquatic photochemical processes.

## Abstract 2

This study explores the photochemical production of hydroxyl radicals (^•^OH) by dissolved organic matter through a H_2_O_2_-dependent pathway, investigating the role of iron in the photo-Fenton reaction and the ^•^OH formation through ketyl radical-driven processes.

## 1 Introduction

Photochemical processes play an important role in controlling the fate of chemicals in surface water and include direct photolysis of sunlight-absorbing molecules and indirect photolysis. Indirect photolysis involves a suite of reactive intermediates such as triplet-state dissolved organic matter (^3^DOM*), superoxide radical anion (O_2_^•–^), hydrogen peroxide (H_2_O_2_), hydroxyl radical (^•^OH), and singlet oxygen (^1^O_2_).^1^ Among these reactive species, ^•^OH plays an important role in environmental oxidation reactions as it reacts nonselectively with a wide range of organic and inorganic compounds at near-diffusion-controlled rates (*k* ∼ 10^9^ M^–1^ s^–1^).^2^ In natural waters, dissolved organic matter (DOM) has been recognized as one of the primary sources of ^•^OH photoproduction,^3,4^ yet the underlying mechanisms remain elusive.

Two ^•^OH formation pathways from DOM photolysis have been proposed: a H_2_O_2_-dependent pathway and a H_2_O_2_-independent pathway.^5−9^ The intermediacy of H_2_O_2_ in the formation of ^•^OH is supported by the suppression of ^•^OH formation with the addition of catalase as a quencher of endogenous H_2_O_2_.^10^ Previous assessments to quantify the contribution of H_2_O_2_ found that the H_2_O_2_-dependent pathway contributes 15–50% of the total formation rate of ^•^OH from various DOM sources, including model DOM compounds,^11^ DOM isolates,^4,12^ wastewater-derived DOM,^13^ and soil DOM.^12,14^ The mechanisms responsible for the H_2_O_2_-independent pathway remain elusive and difficult to quantify. Studies have demonstrated that model compounds like 2,4-dihydroxybenzoic acid can generate ^•^OH, presumably through a process involving the formation of a triplet-state quinoid enol tautomer followed by hydrogen atom abstraction from water.^15^ Additionally, triplet sensitizers such as *p*-benzoquinone have been found to hydroxylate arene probe compounds through yet-to-be fully elucidated mechanisms. The contribution of these “low-energy hydroxylators” complicates our ability to further understand ^•^OH formation mechanisms using arene probes because some (unknown) proportion of the ^•^OH formation rate is not free ^•^OH.^16^ H_2_O_2_-dependent production of ^•^OH can occur through various mechanisms, including the direct photolysis of H_2_O_2_, transition-metal catalysis (Fenton reaction),^4,17−19^ and the reactions of H_2_O_2_ with DOM or other reactive species.^20−23^ Direct photolysis of H_2_O_2_ in natural waters is relatively slow due to minimal overlap between H_2_O_2_ absorbance and the solar spectrum.^24,25^ Production of ^•^OH via the photo-Fenton reaction is important in Fe-rich waters, particularly waterbodies polluted by acid-mine drainage.^26^ The photo-Fenton reaction significantly contributes to ^•^OH production and is most efficient at acidic pH, where abundant Fe remains soluble to cycle between the two oxidation states (II and III).^27−29^ In these processes, Fe(III) exists as the dominant species in the presence of oxygen and forms complexes with DOM. It can be photochemically reduced to Fe(II) under irradiation through ligand-to-metal charge transfer (LMCT), with Fe(II) subsequently reducing H_2_O_2_ to OH^–^ and ^•^OH.^19^

Although previous studies have advanced our understanding regarding the photo-Fenton reaction in natural waters, knowledge gaps still exist concerning the contribution of this pathway toward the total ^•^OH formation in waters containing DOM with varying Fe levels at circumneutral conditions. Fe(II) is more soluble but readily oxidized to Fe(III) by dissolved oxygen, and the resulting concentrations of Fe(II) measured in seawater^30^ and freshwater are generally on the nanomolar scale in aerobic environments.^31−33^ This prompts the question of whether the Fe levels present in different contexts (e.g., DOM isolate solutions and natural waters) are sufficient to catalyze a significant fraction of photochemically produced H_2_O_2_ into ^•^OH. Considering the variability of Fe concentrations in different environmental matrices, understanding the contribution of naturally occurring Fe becomes essential. For example, DOM isolates from the International Humic Substances Society (IHSS) contain ∼ μg/mg_C_ levels of Fe,^34,35^ and it is unclear whether the background Fe levels present in these isolates contribute to ^•^OH formation. Nakatani et al.^36^ measured generally lower rates of ^•^OH production from the photo-Fenton reaction compared to other sources during irradiations of river samples with pH 6.7–9.0. Vione et al.^3^ also suggested that the photo-Fenton has a minor but non-negligible contribution to ^•^OH formation in natural waters with total Fe concentrations less than 1 μM. These observations align with studies indicating that micromolar concentrations of total Fe are necessary in natural waters for Fe(II) to accumulate to levels high enough to act as a significant sink of H_2_O_2_.^37,38^

There is some prior literature evidence of Fe-independent H_2_O_2_-dependent, ^•^OH formation. Zhu et al.^39,40^ documented that ^•^OH generation occurred from nucleophilic attack by H_2_O_2_ on the electron-poor tetrachloro-1,4-benzoquinone. In a study investigating ^•^OH production during the aeration of electrochemically reduced humic substances, Page et al.^10^ noted that the amount of native Fe present in the isolate materials was insufficient to produce the observed ^•^OH levels through the Fenton reaction alone. This indicates that alternative mechanisms, such as organic radical involvement, may play a significant role. Blough and co-workers suggested that DOM irradiation yields a one-electron reducing intermediate (DOM^•–^), which was hypothesized to be a semiquinone-like radical.^41,42^ DOM^•–^ can be photogenerated through intramolecular electron transfer, as exemplified by the ^3^DOM* oxidation of compounds like phenols or anilines.^22^ This DOM^•–^ could then serve as a reductant of O_2_ and/or H_2_O_2_, leading to ^•^OH formation. Using model triplet sensitizers, Sha et al.^20^ reported that DOM^•–^ (and not ^3^DOM*) was the major species resulting in the photodegradation of H_2_O_2_. These findings suggest that the photogenerated reactive species may further reduce H_2_O_2_ to form ^•^OH. Consequently, the role of DOM in the photochemical generation of ^•^OH via the H_2_O_2_-dependent pathway remains insufficiently studied, as previous work has predominantly focused on photo-Fenton processes, leaving the species reaction involving DOM^•–^ underexplored.^4,17,27,37,43^ Furthermore, distinguishing the contribution from H_2_O_2_-dependent and -independent pathways advances our mechanistic understanding and improves models of pollutant fate and transport under diverse environmental conditions.

The objective of this study is to quantify the contribution of the H_2_O_2_-dependent pathway toward ^•^OH production from DOM photolysis, further focusing on the importance of Fe and the photo-Fenton reaction. First, two widely used probe compounds, benzoate and terephthalate, were selected to cross-validate the quantified total ^•^OH formation rate. A diverse suite of DOM sources was examined, including nine isolates from the IHSS, three hydrophobic organic acid (HPOA) isolates, three secondary treated wastewater effluents, and one surface water. Bovine liver catalase was employed to differentiate between the H_2_O_2_-dependent and independent pathways. The influence of the photo-Fenton reaction, under oxic and neutral pH conditions, was also analyzed by examining the relationship between Fe levels and ^•^OH production from the H_2_O_2_-dependent pathway. Alternative reaction pathways involving the ^3^DOM* and DOM^•–^ were evaluated for their participation in producing ^•^OH. Overall, this study demonstrates that H_2_O_2_-independent processes dominate the total ^•^OH formation rate in DOM systems containing low Fe/DOM ratios under oxic circumneutral conditions.

## 2 Materials and Methods

### 2.1 Chemicals and Solution Preparation

DOM isolates obtained from the IHSS included Suwannee River humic acid (SRHA, 3S101H), Suwannee River fulvic acid (SRFA, 3S101F), Suwannee River natural organic matter (SRNOM, 2R101N), Upper Mississippi River natural organic matter (MRNOM, 1R110N), Pahokee Peat humic acid (PPHA, 1S103H), Pahokee Peat fulvic acid (PPFA, 2S103F), Elliott Soil humic acid (ESHA, 5S102H), Elliott Soil fulvic acid (ESFA, 5S102F), and Pony Lake fulvic acid (PLFA, 1R109F). DOM stock solutions (∼200 mg/L) were prepared by dissolving the solid isolate in lab-grade water produced from a Barnstead Nanopure purification system (Thermo Scientific, 18.20 MΩ-cm resistivity). Secondary wastewater effluent was collected at the discharge of Texas A&M University Wastewater Treatment Plant in College Station, Texas. Whole water samples were collected from Town Creek in Palestine, Texas (31°43′16.8″N, 95°41′41.3″W). The effluent and whole water samples were filtered through a 1.50 μm muffled (500 °C, 4 h) glass fiber filter (Whatman GF/F) followed by a 0.45 μm MCE filter (GN-6 Metricel, Pall Corporation) and stored at 4 °C until use. Details of solution preparation are included in the Supporting Information (Text S1).

Experimental solutions were irradiated in uncapped, borosilicate glass tubes placed in a Rayonett merry-go-round photoreactor equipped with mercury vapor lamps of emission maxima at 365 nm (Figure S1). The absolute irradiance of the light source (*I*_0,λ_) was assessed to be 1.49 × 10^–7^ Es cm^–2^ s^–1^ by actinometry based on the *p*-nitroanisole/pyridine method.^44^ To determine the formation rate of ^•^OH, irradiations were carried out with solutions containing the photosensitizer and a probe compound (benzoate or terephthalate) at a range of concentrations (10–200 μM). H_2_O_2_ (200 μM) and nitrite (NO_2_^–^, 230 μM) were employed as the model sensitizers in the absence of DOM to compare the performance of the two probes. DOM was used at a concentration of 20 mg/L unless otherwise specified. To maintain a pH of 7, solutions were buffered with 10 mM phosphate. Catalase concentrations between 0 and 40 units/mL were used as documented in Text S1.

### 2.2 Instrumental Analysis

Hydroxylated probe compounds (salicylate and hydroxyterephthlate) were monitored through ultrahigh-performance liquid chromatography (UHPLC, Thermo Fisher) with a fluorescence detector. Inductively coupled plasma mass spectrometry (ICP-MS, PerkinElmer NexION 300D) was used to measure the total Fe concentration in DOM solutions. For whole water samples, ion analysis was conducted on a Dionex Integrion ion chromatograph equipped with a conductivity detector. Total organic carbon (TOC) was measured by a Total Organic Carbon Analyzer (TOC-L series, Shimadzu). Further details on the instrumental analysis are provided in Text S3.

### 2.3 Computational Methods

The one-electron reduction potentials (*E*^0^) of acetophenone, *p*-benzoquinone, and H_2_O_2_ were calculated using density functional theory on the Gaussian 16 platform.^45^ The acid association constant (p*K*_a_) of radicals was also calculated with the same level of theory to discuss the possibility of reducing radical anion protonation at pH 7. Details are included in Text S6.

## 3 Results and Discussion

### 3.1 Hydroxyl Radical Formation Rates (*R*_OH_)

Use of benzoate and terephthalate to determine the ^•^OH formation rate (*R*_OH_) relies on the accuracy of the formation yield of the hydroxylated probe compound (Text S4). In contrast to many prior studies that monitored *p*-hydroxybenzoate from benzoate hydroxylation,^46−51^ salicylate (*o*-hydroxybenzoate) was selected in this study as it is strongly fluorescent (Figure S3). However, the yield of salicylate has not been extensively reported. Sun et al.^48^ reported a salicylate yield ranging from 0.17 to 0.18 without indicating a derivation method. In the study by Zhou and Mopper,^52^ the isomer ratios of *o*-, *m*-, and *p*-hydroxybenzoate were measured as 36, 34, and 30%, respectively, and the reaction yield of *p*-hydroxybenzoate was determined as 0.17 from a two-probe intercalibration (methanol and benzoate). In our work, by establishing a relationship between the isomer ratio and the isomer yield within the same system (Text S5), we calculated the yield of salicylate to be 0.204. A similar yield calculation approach was employed by Anastasio et al.,^46^ who derived a yield of 0.19 for *m*-hydroxybenzoate.

Benzoate and terephthalate were applied at a range of concentrations from 20 μM to 200 μM to quantify *R*_OH_ using the method described by eqs S4–4b (Text S4). H_2_O_2_ (200 μM) and NO_2_^–^ (230 μM), serving as model sensitizers for ^•^OH production, were irradiated to assess the performance of the probes. As shown in Table 1, *R*_OH_ values obtained by both probes during photolysis of H_2_O_2_ were in good agreement, with benzoate measuring (0.538 ± 0.006) × 10^–9^ M/s and terephthalate (0.577 ± 0.016) × 10^–9^ M/s. Similar results were obtained, utilizing NO_2_^–^ as a ^•^OH radical source, with the *R*_OH_ measured being (32.2 ± 6.2) × 10^–9^ M/s by benzoate and (32.9 ± 7.4) × 10^–9^ M/s by terephthalate (Figures S4 and S5). Thus, both benzoate and terephthalate provided consistent measurements of *R*_OH_.

**Table 1:** Summary of Hydroxyl Radical Formation Rates (*R*_OH_) Measured from Benzoate and Terephthalate in H_2_O_2_- and NO_2_^–^-Sensitized Systems

probe	benzoate	terephthalate
Hydroxylated product	salicylate	hydroxyterephthalate
Yield	0.204^a^	0.35^53^
*k*_2_ (×10^9^ M^–1^ s^–1^)^b^	5.9^54^	4.4^12^
*R*_OH_ (×10^–9^ M/s) by 200 μM H_2_O_2_	0.538 ± 0.006	0.577 ± 0.016
*R*_OH_ (×10^–9^ M/s) by 230 μM NO_2_^–^	32.2 ± 6.2	32.9 ± 7.4

a The yield of salicylate was determined in this work (Text S5).

b *k*_2_ corresponds to the second-order rate constant (M^–1^ s^–1^) between the probe and ^•^OH (Text S4).

**Figure 1:**
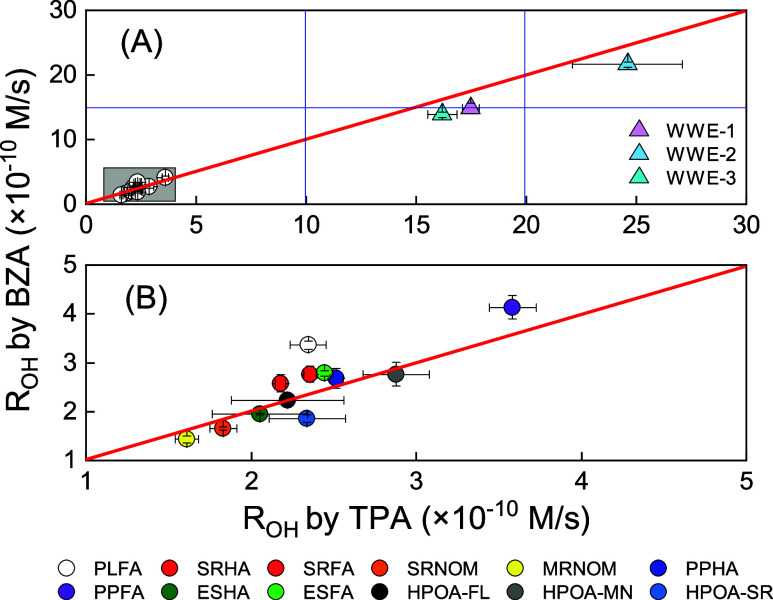
Assessment of the hydroxyl radical formation rate (*R*_OH_) under UV irradiation (365 nm) in a 20 mg/L DOM-sensitized system. *R*_OH_ measured by terephthalate (TPA) is plotted on the *x*-axis and *R*_OH_ measured by benzoate (BZA) on the *y*-axis. The top panel (A) displays all data, while the bottom panel (B) emphasizes samples with lower *R*_OH_. The red diagonal line represents the 1:1 ratio.

We continued to employ benzoate and terephthalate for the measurement of *R*_OH_ using DOM as a sensitizer. Figure 1 demonstrates the correlation between *R*_OH_ values obtained from the two probes for 12 DOM isolate materials and three wastewater effluents. DOM isolates exhibited *R*_OH_ values between 1.5 and 3.6 × 10^–10^ M/s at a concentration of 20 mg/L. Wastewater effluent samples exhibited higher *R*_OH_ than DOM solutions (>1 × 10^–9^ M/s). This can be attributed to their high nitrate (NO_3_^–^) concentrations (∼40 mg/L, Table S2), as NO_3_^–^ is likely to prevail as the ^•^OH source when the NO_3_^–^/TOC ratio is above 2.0 mg NO_3_^–^/mg_C_.^3^ Generally, most data closely align with the 1:1 line, reflecting consistent *R*_OH_ values measured with benzoate and terephthalate. PPFA and PLFA are slightly off this trend, both exhibiting higher *R*_OH_ measured by benzoate compared to terephthalate. This could indicate that benzoate is more susceptible to oxidation by low-energy hydroxylating species produced by PLFA and PPFA. Page et al.^12^ also suggested that photohydroxylation of terephthalate by PLFA was due, in part, to low-energy hydroxylators. In contrast, ESHA was also reported to have low-energy hydroxylators based on methane quenching, but in our study, *R*_OH_ values measured by benzoate and terephthalate were in good agreement. Despite these differences, the variations were relatively small (within a factor of 1.5 or less), suggesting that benzoate and terephthalate provide similar estimates of *R*_OH_.

### 3.2 Apportionment of H_2_O_2_-Dependent and H_2_O_2_-Independent Pathways by Catalase

Extensive prior literature has employed catalase to decompose H_2_O_2_ as a precursor to ^•^OH radical, including studies focused on ^•^OH production from DOM.^4,11,12,55−57^ In principle, measuring ^•^OH formation while suppressing H_2_O_2_ with catalase allows the contribution of H_2_O_2_ to the total ^•^OH production to be calculated. However, in preliminary investigations conducted with 10 μM benzoate as a probe, we observed that ^•^OH quenching by catalase was comparable to that of the background and the probe. ^•^OH quenching by catalase could cause the contribution of the H_2_O_2_-dependent pathway (reported in prior literature to be up to 50%) to be overpredicted.^12^ Control experiments (Text S7) utilizing NO_2_^–^ as a ^•^OH source demonstrated that the extent of ^•^OH scavenging by catalase could be minimized (<2.4%) by using 2 mM benzoate as a probe and catalase concentrations less than 50 units/mL. Experiments (Figure S7) also derived a catalase ^•^OH scavenging rate constant as 2900 ± 200 mL unit^–1^ s^–1^, corresponding to a scavenging rate of ∼1.45 × 10^5^ s^–1^ for 50 units/mL catalase. Using an ^•^OH reaction rate constant with organic carbon of 1.9 × 10^4^ L mg_C_^–1^ s^–1^, the addition of 50 units/mL catalase contributed a TOC of 8.95 ± 0.24 mg_C_/L (Figure S8). This amount of TOC resulted in a calculated ^•^OH scavenging rate of 1.70 × 10^5^ s^–1^.^58,59^ These values show strong agreement and quantitatively support the use of 2 mM benzoate with a catalase concentration less than 50 units/mL to minimize catalase scavenging of ^•^OH.

**Figure 2:**
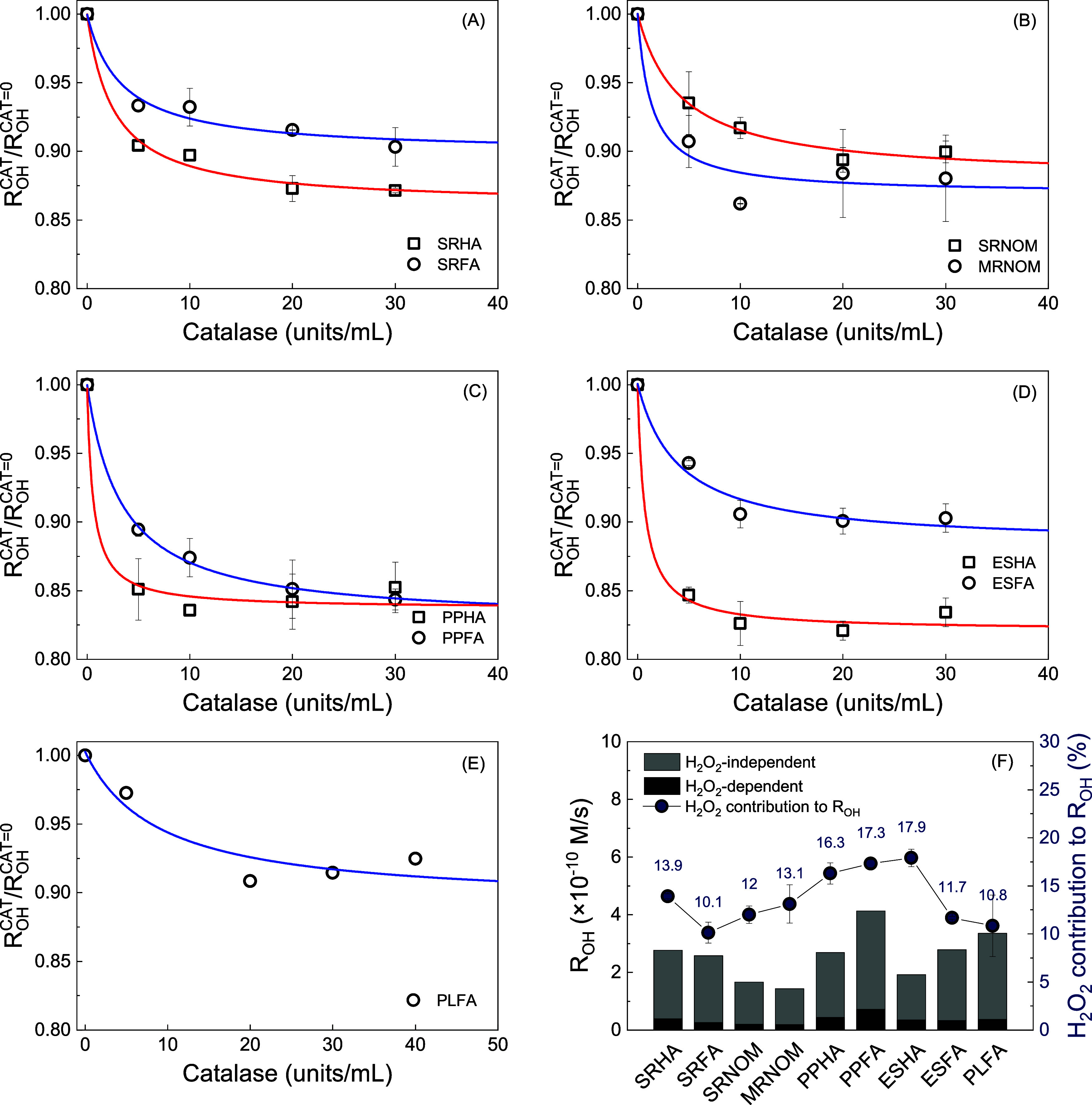
Catalase experiment with a benzoate concentration of 2 mM at catalase concentrations between 0 and 40 units/mL. A DOM concentration of 20 mg/L was used for isolates including (A) SRHA and SRFA, (B) SRNOM and MRNOM, (C) PPHA and PPFA, (D) ESHA and ESFA, and (E) PLFA. *R*_OH_^CAT^/*R*_OH_^CAT=0^ on the *y*-axis represents the relative rate of ^•^OH formation with versus without the addition of catalase. (F) Formation rate of ^•^OH (*R*_OH_) from the H_2_O_2_-dependent pathway and the H_2_O_2_-independent pathway determined through applying a catalase quenching experiment. Averaged duplicate data points were obtained from the catalase quenching experiment, and the solid lines were modeled fitting results developed by Page et al.^12^

Using these conditions, we measured *R*_OH_ as a function of catalase concentration using nine isolated materials from the IHSS. Figure 2A displays the results from SRHA and SRFA following the fitting model developed by Page et al.^12^ Based on the model fitting results, *R*_OH_ was quenched by 13.9 ± 0.3% for SRHA and 10.1 ± 1.1% for SRFA. SRNOM (Figure 2B) demonstrated a catalase quenching of 12.0 ± 0.9%, which closely parallels the results from SRHA and SRFA. PPFA and ESHA exhibited the highest catalase quenching values of 17.3 ± 0.4 and 17.9 ± 0.9%, respectively. While previous research using PLFA did not observe significant quenching of *R*_OH_ by catalase,^12^ our experiments revealed that *R*_OH_ from PLFA was quenched by 10.8 ± 3.2% (Figure 2E). This discrepancy may have been due to the substantial ^•^OH scavenging by 40 units/mL catalase (∼1.1 × 10^5^ s^–1^) under the 10 μM concentration of terephthalate (∼4.4 × 10^4^ s^–1^) employed previously.^12^ No catalase quenching was observed for secondary wastewater effluents, likely because ^•^OH production was dominated by NO_3_^–^ photolysis (H_2_O_2_-independent; Figure S9).

Based on the attenuation of *R*_OH_ with increasing concentrations of catalase, we were able to determine the contribution of H_2_O_2_-dependent and independent pathways to ^•^OH formation. As summarized in Figure 2F, the H_2_O_2_-dependent pathway contributed 10–20% of the total *R*_OH_ for DOM isolates of diverse sources (aquatic vs soil) and isolation procedures (reverse osmosis vs XAD). Thus, for IHSS isolates, which are among the most widely studied sample types in DOM photochemistry, the H_2_O_2_-dependent pathway constitutes only a small fraction of the total ^•^OH formation, with the H_2_O_2_-independent pathway predominating. The catalase quenching effect observed in this study is generally lower than those reported in previous studies, even those focused on isolate materials. For example, Vaughan and Blough observed at least a 50% reduction in *R*_OH_ due to catalase quenching in SRFA under a 310 nm UV light using 10 mM dimethyl sulfoxide as a probe.^4^ In contrast, Page et al.^12^ reported an approximate 15% H_2_O_2_ contribution for SRHA and SRFA. Wan et al.^11^ evaluated the photogeneration of ^•^OH from H_2_O_2_ sensitized by hydroxybenzoic acid; in that study, the formation rate of phenol from a 3 mM benzene probe was reduced by 53% at 100 units/mL catalase. In the same study, using DOM as a ^•^OH sensitizer, a 62% reduction in ^•^OH formation was observed after the addition of 100 units/mL of catalase.^11^ Furthermore, the study by Yu et al.^60^ employed 1000 units/mL addition of catalase to DOM reduced with dithionite and observed the decrease of ^•^OH production of 31%. Taken together, the contribution of the H_2_O_2_-dependent pathway to the formation of ^•^OH reported in the prior literature ranges from 10 to 50%. Beyond differences in the DOM composition that may contribute to this variability, it is important to emphasize the need for careful experimental controls when interpreting results from catalase quenching experiments, as catalase acts as a quencher for both H_2_O_2_ and ^•^OH radicals.

### 3.3 Apportionment of Photo-Fenton Reaction to H_2_O_2_-Dependent Pathway

**Figure 3:**
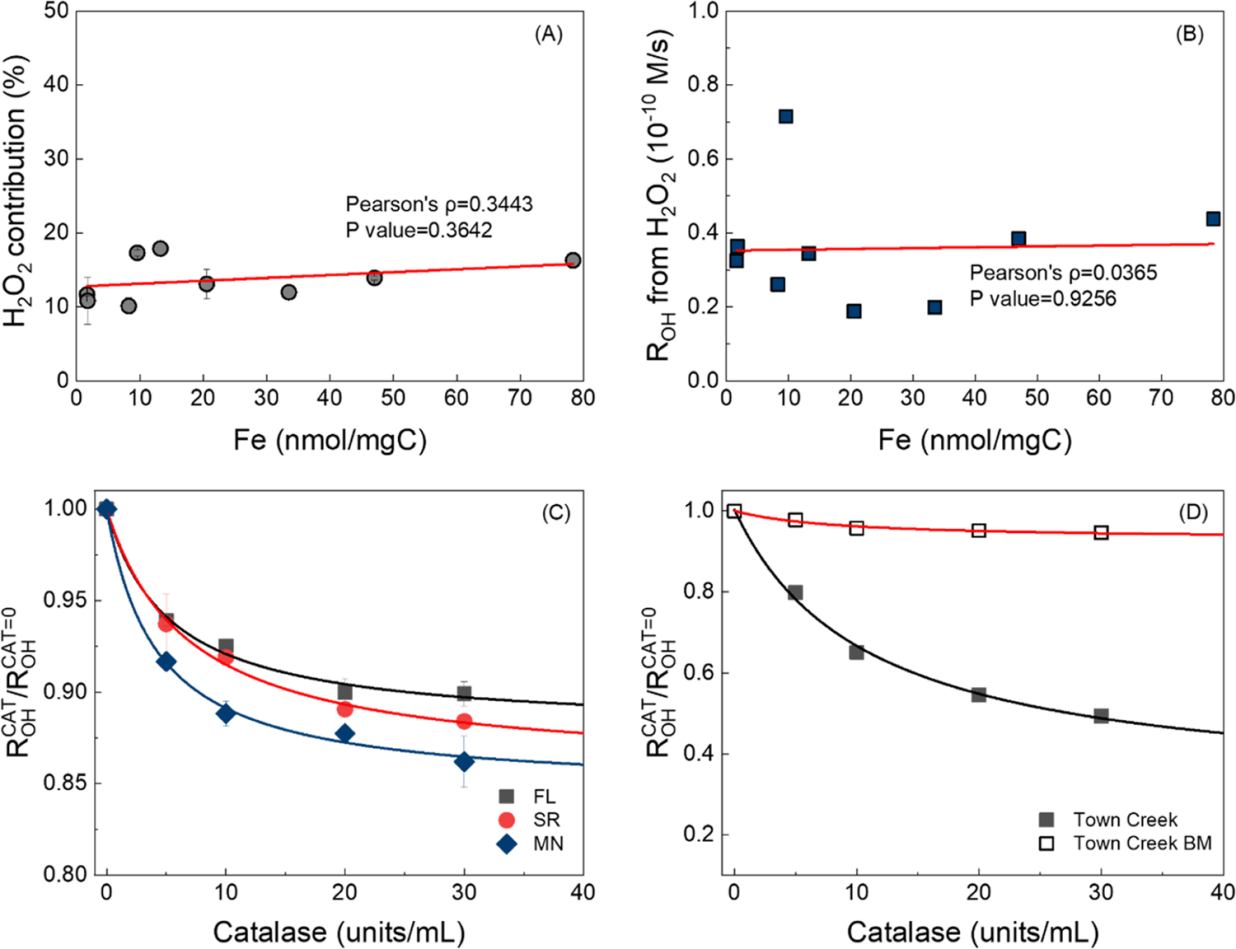
(A) Correlation between Fe concentration and the contribution in the H_2_O_2_-dependent pathway. (B) Correlation between Fe concentration and ^•^OH formation from the H_2_O_2_-dependent pathway. (C) Catalase experiment with HPOA isolates at 20 mg/L using 2 mM benzoate. Isolates from Florida (FL), Suwannee River (SR), and Minnesota (MN) exhibited varying total Fe concentrations. (D) Catalase experiment with Town Creek water and base-modified Town Creek water (Town Creek BM) using 2 mM benzoate.

Correlations were evaluated between Fe concentration and the contribution of the H_2_O_2_-dependent pathway to *R*_OH_. Figure 3A illustrates a moderate positive correlation between Fe concentrations and the H_2_O_2_ contribution to ^•^OH formation; however, the p-value (0.3642) suggests that the relationship is not statistically significant. Similarly, Figure 3B suggests no significant relationship between the Fe concentration and H_2_O_2_-dependent *R*_OH_. These results indicate that Fe concentration does not significantly influence the formation rate of ^•^OH through the H_2_O_2_-dependent pathway under the tested conditions (ambient Fe levels, an oxic environment, and neutral pH). Notably, PPHA, despite having a much higher Fe concentration than PPFA (78.3 vs 9.6 nmol Fe/mg_C_), exhibited a similar attenuation of *R*_OH_ (16.3 ± 1.1 vs 17.3 ± 0.4%). Even with low Fe levels in ESFA and PLFA (1.6 and 1.8 nmol Fe/mg_C_, respectively), there was an ∼10% contribution from the H_2_O_2_ pathway.

To further investigate the relationship between Fe and the H_2_O_2_-dependent pathways, three HPOA isolates with varying total Fe concentrations were studied. HPOA-SR and MN contained 45.0 and 202.0 nmol of Fe/mg_C_, respectively, while the Fe content in HPOA-FL was below the detection limit. Despite the large variance in the Fe content, the difference in the catalase quenching effect was relatively minor (∼5%). Specifically, HPOA-MN showed a catalase quenching effect of 15.4 ± 0.7%, followed by HPOA-SR at 14.4 ± 0.8% and HPOA-FL at 12.1 ± 0.8%, despite its undetectable Fe levels. In addition, we analyzed whole water samples with the catalase quenching experiment. Town Creek water (Figure 3D) revealed a quenching effect of up to 50% with catalase added at 40 units/mL. Fitting results predicted a contribution of 69.9 ± 4.8% from H_2_O_2_ to the total ^•^OH formation. ICP-MS analysis measured an Fe concentration of 1073.5 ± 13.2 μg/L (1970.8 nmol of Fe/mg_C_) in Town Creek, which is 10–1000 times higher than the Fe content in reference DOM and HPOAs. To evaluate the role of Fe, base modification (Text S1) was performed to remove metals from Town Creek water, decreasing the Fe concentration to 66.1 ± 2.9 μg/L (139.6 nmol Fe/mg_C_).^61,62^ In contrast to the extensive quenching of *R*_OH_ observed in native Town Creek water, the base-modified sample had a markedly decreased attenuation in *R*_OH_ (7.0 ± 0.9%). The concomitant decrease in Fe concentrations and lack of *R*_OH_ attenuation after base modification corroborate catalase quenching as a tool for quantifying the H_2_O_2_-dependent pathway and further suggest that Fe must be present at sufficiently high concentrations for the photo-Fenton reaction to play a significant role in ^•^OH formation. Overall, these results indicate that pathways besides the photo-Fenton reaction predominate in the DOM systems at lower Fe concentrations, a conclusion consistent with other studies. For example, Nakatani et al.^36^ observed a poor correlation between the photo-Fenton ^•^OH formation rate and total Fe (<2 μM). Similarly, Page et al.^43^ found that the contribution of Fe to ^•^OH formation through the photo-Fenton reaction for filtered Arctic whole water samples does not increase in proportion to the Fe concentration.

The photo-Fenton reaction rate depends on the concentrations of H_2_O_2_ and Fe(II), and rate constants for the reactions involved in the process, such as the photoreduction of Fe(III), and the Fenton reaction under specific pH conditions.^30,63^ To evaluate the impact of H_2_O_2_ concentration, we measured H_2_O_2_ formation rates for DOM isolates under identical conditions (DOM concentration, pH, light source, irradiation intensity, and irradiation time). The formation rates ranged from 0.72 nM/s for MRNOM to 2.51 nM/s for ESHA (Figure S10). Therefore, H_2_O_2_ concentrations in DOM solutions under UV irradiation are 1–2 orders of magnitude higher than Fe concentrations, indicating that the rate-limiting step for ^•^OH production is the photoreduction of Fe(III) to Fe(II). In oxic environments, Fe exists in DOM mainly in the form of Fe(III)–DOM complexes. Two major pathways account for the photochemical reduction of Fe(III): LMCT and O_2_^•–^-mediated Fe reduction. Le Roux et al.^23^ demonstrated that metal chelators (to inhibit the reaction of metals with reactive species) had no impact on O_2_^•–^ formation and therefore posited a minimal role of O_2_^•–^ in Fe(III) photoreduction. This, along with previous studies reporting rapid Fe(III) reduction by LMCT,^64−66^ indicates that the rate of ligand-to-metal charge transfer is a key process determining the concentration of Fe(II) available to generate ^•^OH via reaction with H_2_O_2_.

Kinetic modeling (Text S8) was used to assess the photo-Fenton reaction, focusing on Fe cycling reactions. The Fe(III) photoreduction rate constant varies based on several extrinsic factors, with reported values ranging from *k*_LMCT_ = 10^–3^ to 10^–2^*s*^–1^.^19,66^ Sensitivity analysis showed that for SRHA, the concentration of Fe(II) calculated using eq S8–5 increased from 15.8 nM to 213 nM as *k*_LMCT_ increased from 10^–3^ to 10^–2^ s^–1^ (Figure S11), which could subsequently enhance the production of ^•^OH through the Fenton reaction. Similar analyses for other DOM isolates revealed a 6- to 13-fold increase in Fe(II) concentrations. The variability in LMCT remains unknown among different DOM isolates as it is influenced by DOM composition, the type of complexing ligand, and light intensity and wavelength.^67,68^ Studies using environmental samples demonstrate that DOM from different sources has varied effects on Fe redox cycling. At neutral pH, the majority of Fe is complexed to carboxyl groups in DOM, such as citrate, catechol, EDTA, and bipyridine-like complexes.^69^ A study by Hudson et al. showed that reduction potentials of Fe(III) complexes are highly dependent on the ligand type, with acetate being the highest at 0.653 V and EDTA the lowest at 0.109 V.^70^ Differences in DOM composition can result in variations in the type of Fe complexing ligands, which, in turn, can influence the redox potential and LMCT processes under irradiation. For example, the reduction potential of Fe(III) was measured to be 0.071 V for SRHA, 0.043 for SRFA and SRNOM, and 0.067 V for PLFA.^70^ In addition, pH changes can alter the number of Fe binding sites,^70^ further increasing the uncertainty in LMCT efficiency and the subsequent production of ^•^OH from the Fenton reaction.

Collectively, ^•^OH production from the photo-Fenton reaction is controlled by several factors, and similar Fe levels in different DOM isolates do not consistently yield comparable ^•^OH production. As estimated by Vermilyea and Voelker,^29^ the photo-Fenton reaction contributed around 26 ± 13% of total ^•^OH production at neutral pH, with the lower end aligning with our findings. Overall, our study confirms the photo-Fenton reaction as a source of ^•^OH but with lower efficiency at ambient concentration levels than previously reported.^17,29,71^

### 3.4 Participation of ^3^DOM* and DOM^•–^ in the H_2_O_2_-Dependent Pathway

Our results indicate that the photo-Fenton reaction is a minor contributor to ^•^OH production from the H_2_O_2_-dependent pathway for DOM samples with lower Fe contents (<200 nmol Fe/mg_C_) (e.g., isolates, low Fe waters). As illustrated in Figure S10, UV irradiation significantly influences the production and attenuation of H_2_O_2_. H_2_O_2_ levels in SRHA and SRFA solutions increased to 6.3 and 3.5 μM, respectively, after 60 min of irradiation. In the absence of light, H_2_O_2_ levels remained stable for several hours (<20% loss in 24 h for SRHA), but no dark formation of hydroxylated probe products was observed (Figure S12). This observation is consistent with the limited impact of dark Fenton reactions due to the low total Fe concentration present in DOM isolate solutions. However, the excess H_2_O_2_ existing under irradiation conditions could readily serve as an electron acceptor to form ^•^OH. This prompted further investigation into alternative mechanisms involving H_2_O_2_.

**Figure 4:**
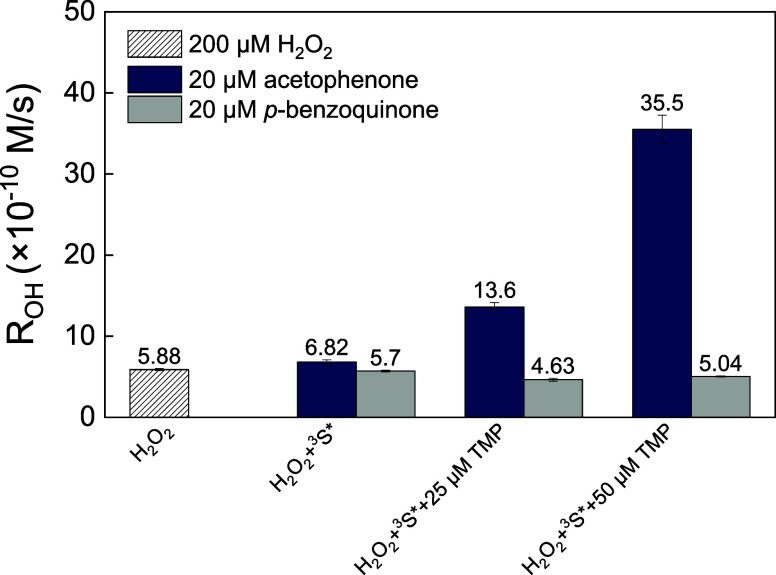
*R*_OH_ from the reaction between 200 μM H_2_O_2_ and other reactive species. The gray column with slanted stripes represents the baseline *R*_OH_ produced solely by 200 μM H_2_O_2_. The blue column indicates the *R*_OH_ when acetophenone is used as a sensitizer at 20 μM, and the solid gray column shows the *R*_OH_ with 20 μM *p*-benzoquinone as the sensitizer. Both sensitizers are depicted in their triplet states (^3^S* in the *x*-axis) under UV_365_ irradiation. Additionally, 2,4,6-trimethylphenol (TMP) was tested at concentrations of 25 and 50 μM to observe its effect on the *R*_OH_ production.

^3^DOM* is known to act as both an oxidant and a reductant,^72^ and recent studies have demonstrated the ability of ^3^DOM* to reduce halogenated organics,^73^ chlorine disinfectants,^74,75^ and peroxymonosulfate.^76^ To evaluate the role of ^3^DOM* and H_2_O_2_ in ^•^OH formation, we employed two model triplet photosensitizers, acetophenone (ACP) and *p*-benzoquinone (*p*BQ), which represent aromatic ketone and quinone groups prevalent in DOM. Control experiments (Figure S13) indicated no ^•^OH formation during photolysis of acetophenone alone and a low formation rate (4.66 × 10^–12^ M/s of hTPA) for *p*-benzoquinone using terephthalate as a probe to measure ^•^OH. Acetophenone and *p*-benzoquinone (20 μM) were subsequently irradiated in the presence of 200 μM H_2_O_2_. The presence of acetophenone or benzoquinone triplets did not enhance the *R*_OH_ beyond what was observed with H_2_O_2_ alone (Figure 4). This result is consistent with prior research demonstrating that triplet sensitizers do not enhance H_2_O_2_ photodegradation.^74,77^

Although triplet sensitizers were unreactive toward H_2_O_2_, their one-electron reduction products serve as another potential reductant (Sens^•–^). One-electron reduction products form upon the reaction of excited triplet states with electron-rich moieties, such as phenols and anilines,^22^ and were suggested to be involved in H_2_O_2_ photodegradation.^20^ To evaluate the role of Sens^•–^ in ^•^OH production via reduction of H_2_O_2_, we introduced TMP to acetophenone and *p*-benzoquinone solutions containing H_2_O_2_. Enhanced ^•^OH photoproduction was observed in the solution with acetophenone as the sensitizer (Figure 4). For example, adding 25 μM TMP increased *R*_OH_ to 2.3 times relative to H_2_O_2_ direct photolysis. Increasing the TMP concentration to 50 μM led to a 6-fold increase in *R*_OH_, suggesting that the addition of TMP promotes the formation of Sens^•–^ and, subsequently, the reduction of H_2_O_2_ to ^•^OH. Previous studies indicated that phenol moieties are critical for the formation of Sens^•–^, and the observation of the TMP concentration increase in this study agrees with a positive correlation between the phenolic content of DOM and H_2_O_2_ photodegradation reported previously.^20^ Conversely, our results showed no *R*_OH_ enhancement with the addition of TMP to *p*-benzoquinone.

The generation of ^•^OH from the acetophenone–TMP system is hypothesized to arise from two primary sources. First, H_2_O_2_ production from the reaction between triplet acetophenone and TMP: acetophenone in its triplet state can react with TMP to produce H_2_O_2_, which subsequently undergoes direct photolysis to produce ^•^OH.^78^ As evidenced by the control experiment (Figure S13), H_2_O_2_ formed from 20 μM triplet acetophenone with 50 μM TMP contributes to merely 5.3% of the *R*_OH_ compared to 200 μM H_2_O_2_ direct photolysis. Therefore, this source does not fully explain the increased *R*_OH_. Second, triplet acetophenone reduction to ketyl radicals: acetophenone in its triplet state acts as a stronger oxidant, with a one-electron reduction potential increased by 3.19 V relative to its ground state (−1.42 to 1.77 V vs SHE from literature values; Table S5).^21,72,79^ Triplet acetophenone could oxidize TMP to yield ketyl radicals and radical anions that are capable of reducing H_2_O_2_ to ^•^OH.

**Figure 5:**
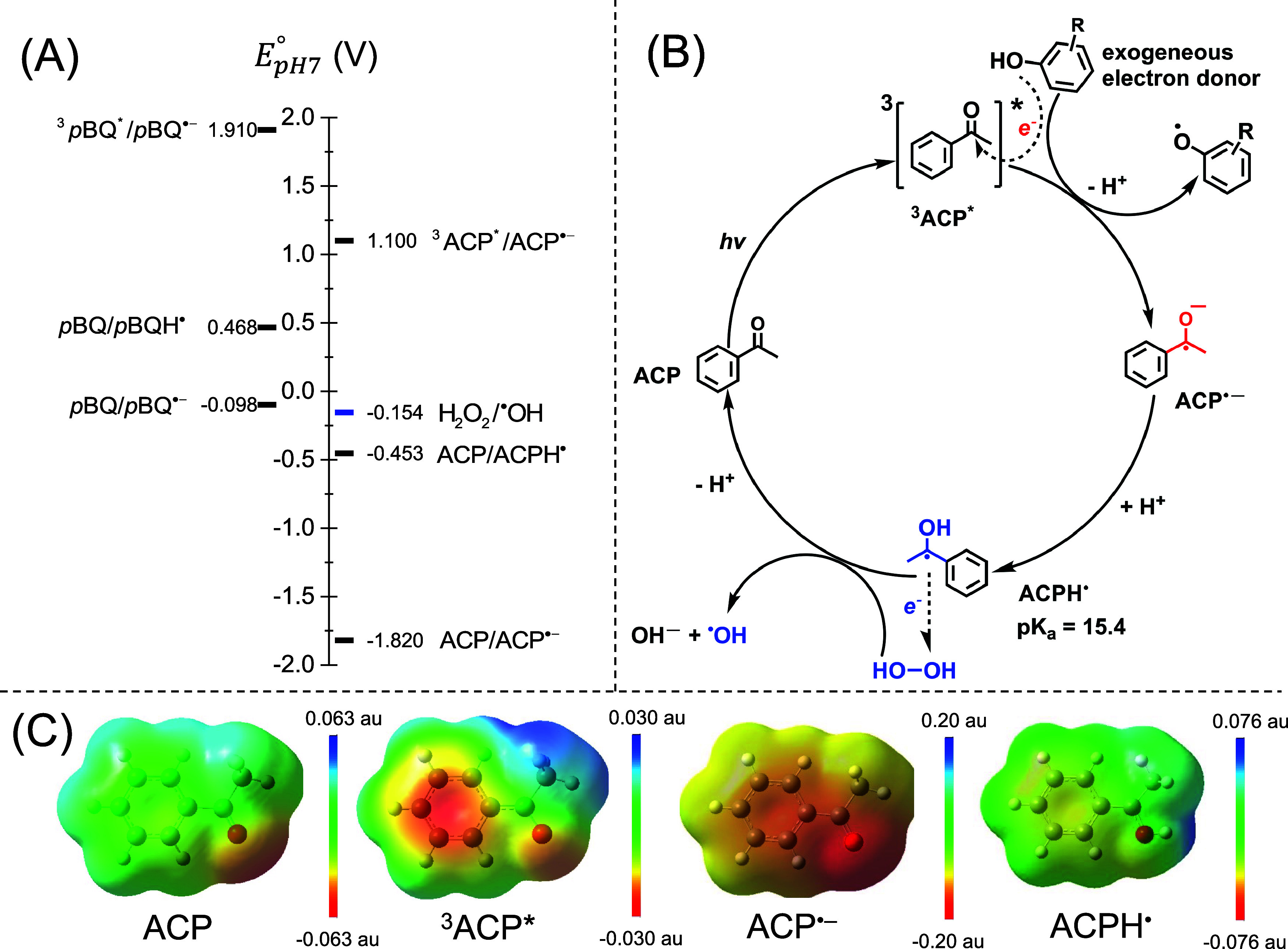
(A) Ground-state (*E*_pH7_^°^) and triplet-state (*E*_pH7_^°^*) reduction potentials at pH 7 for two DOM model sensitizers (acetophenone and *p*-benzoquinone). (B) Mechanism of ^•^OH formation involving the ketyl radical and its reaction with H_2_O_2_. (C) Total electron density cubes with the electrostatic potential (ESP) surface modeling of aqueous phase molecules. Colormap end values are adapted to the min/max ESP of individual compounds. Red-yellow: negative; green: neutral; cyan-blue: positive. Acronyms used in this figure include acetophenone (ACP), triplet acetophenone (^3^ACP*), ketyl radical anion (ACP^•–^), ketyl radical (ACPH^•^), *p*-benzoquinone (*p*BQ), triplet *p*-benzoquinone (^3^*p*BQ*), semiquinone radical anion (*p*BQ^•–^), and semiquinone radical (*p*BQH^•^).

As literature values were obtained under varying experimental conditions—such as the reduction potential for H_2_O_2_/^•^OH, which has been reported to range from 0.39 to 0.46 V^80,81^—and due to a lack of knowledge on the speciation of radicals and radical anions, we performed molecular simulations to calculate the one-electron reduction potential of the reactants. As depicted in Figure 5B, triplet acetophenone accepts one electron from an exogenous phenol donor, forming a ketyl radical anion (ACP^•–^). The electrostatic potential on the ketyl group of ACP^•–^ is 3 times higher than that of ground-state acetophenone, making it more nucleophilic and susceptible to proton attacks in aqueous solution (Figure 5C). Based on the calculated p*K*_a_ of the ketyl radical (ACPH^•^) being 15.4, ACP^•–^ is quickly protonated to form ACPH^•^ at neutral pH. The singly occupied molecular orbital electron in the protonated ACPH^•^ radical is more delocalized in the aromatic ring, instead of only in the ketyl group in the ACP^•–^ species. The electrostatic potential also becomes less negative after protonation. Although ACPH^•^ is less reducing than its anionic form, with its reduction potential *E*_pH7_^°^ (ACP/ACPH^•^) increasing to −0.453 V vs SHE, it remains sufficiently reducing to thermodynamically favor the reduction of H_2_O_2_ to ^•^OH (*E*_pH7_^°^(H_2_O_2_/^•^OH) = −0.154 V vs SHE) (Figure 5A). The Gibbs free-energy change for the electron-transfer reaction (Δ*_r_G*_et_^pH=7^) was calculated to be −28.85 kJ/mol. Triplet *p*-benzoquinone is a stronger oxidant than triplet acetophenone. The semiquinone radical anion (*p*BQ^•–^) formed by electron transfer from an exogenous electron donor has a calculated one-electron reduction potential (*E*_pH7_^°^(*p*BQ/*p*BQ^•–^) = −0.098 V vs SHE), with its protonated radical having an even higher reduction potential (0.468 V vs SHE). The more positive reduction potentials for *p*BQ^•–^ and *p*BQH^•^ suggest that these species are less reactive toward reducing H_2_O_2_ to ^•^OH, which agrees with the observation of an unchanged *R*_OH_ for *p*-benzoquinone irradiated in the presence of H_2_O_2_ and TMP. Although there are reports of Fe-independent production of ^•^OH through the reaction of H_2_O_2_ with halogenated quinones, this reaction was not observed for nonhalogenated quinones.^39,40^ This indicates that electron-withdrawing substituents on the quinone ring might be necessary for the reaction to occur. Interestingly, while quinones did not facilitate ^•^OH generation in this context, they were shown to enhance the rate of ascorbate-mediated reduction of H_2_O_2_.^82^ Overall, the role of quinones in the reduction of H_2_O_2_ and their potential contribution to ^•^OH formation warrant further investigation.

## 4 Environmental Implication

The results presented in this study have significant implications for the photochemical production of ^•^OH via a H_2_O_2_-dependent pathway in natural aquatic systems. Catalase experiments revealed a variable but generally low contribution of H_2_O_2_ to ^•^OH formation from DOM isolates with low ambient Fe contents (<200 nmol Fe/mg_C_), accounting for 10–20% of *R*_OH_ in the tested isolate materials. In contrast, the H_2_O_2_-dependent pathway was directly linked to Fe content (via base modification) for surface water with an elevated Fe content (∼2000 nmol Fe/mg_C_). These results indicate that for DOM samples with lower Fe contents, the H_2_O_2_-dependent pathway for ^•^OH formation primarily involves reduction processes driven by electron-donating species. Upon irradiation, at least two pathways can lead to ^•^OH formation from H_2_O_2_: (1) the photo-Fenton reaction and (2) reduction reactions by organic radicals. For most tested samples, it is unlikely that Fe (below 200 nmol/mg_C_) contributes significantly to ^•^OH formation through photo-Fenton reactions under the conditions studied (irradiation wavelengths, oxic, and circumneutral pH).^3,43,71^ Town Creek water without base modification was an exception, due to its Fe concentration being approximately 10–1000 times higher than those of the other samples. The production of ^•^OH from ketyl radicals formed via the reaction between TMP and ^3^ACP* suggests the involvement of organic radical reduction. The electron-donating capacity (EDC) of DOM is a predetermined property by its molecular composition and closely associated with its phenolic content.^83^ EDC could potentially serve as an indicator of ^•^OH production, similar to how quantum yields of ^1^O_2_ have been shown to correlate negatively with EDC in several DOM and whole water samples.^71,84,85^ Although a study reported a positive relationship between the phenolic content and H_2_O_2_ phototransformation,^20^ there is conflicting information regarding the relationship between ^•^OH quantum yield and EDC. Although McKay et al.^86^ and Berg et al.^71^ found no correlation between EDC and ^•^OH quantum yields for native DOM samples, Sharpless et al.^84^ reported a positive correlation between these variables for three humic substance isolates photooxidized in laboratory experiments, resulting in decreased EDC and ^•^OH quantum yields with increasing irradiation. The relatively low and narrow range of ^•^OH production via each formation pathway further complicates the establishment of robust correlations.

Carboxyl groups are known to dominate among the wide range of structural moieties in DOM. However, ketone and aldehyde functionalities are more reactive and strongly influence the optical properties of DOM.^87^ This work demonstrated that ketone functional groups resembling acetophenone may participate in the reduction of H_2_O_2_ to ^•^OH, whereas quinones like *p*-benzoquinone do not. A recent study by Mitschke et al.^88^ detected up to 30% of the molecular formulas in SRNOM-contained isomers with ketone functionalities. This finding underscores the potential for ketyl radicals to form in the DOM system and suggests the possibility of their contribution being underestimated in such environments. Future research should aim to elucidate the molecular-level characteristics of DOM—such as the types and amounts of triplet-state DOM species and their redox properties—to better understand their roles in ^•^OH formation. Studies employing electronically and sterically diverse model triplet states could also be informative. Moreover, a considerable portion of ^•^OH production occurs independently of H_2_O_2_ through as-yet unknown mechanisms. Further research is needed to uncover the full spectrum of the ^•^OH formation reactions.

## Supporting Information Available

Analytical methods, kinetic modeling methods, derivation of salicylate yield, computational method for one-electron reduction potential, evaluation of catalase duality (text); characterization of water samples, hydroxyl radical formation rates, iron content, ground-state/triplet-state reduction potential of DOM model sensitizers and their electrostatic potential (table); absolute spectral irradiance of UV lamps, control experiments, determination of hydroxyl radical formation, H_2_O_2_ concentration profiles (figure); and reaction kinetic illustration (scheme) (PDF)
