# Supplementary material for: Probing the Photochemical Formation of Hydroxyl Radical from Dissolved Organic Matter: Insights into the H2O2-Dependent Pathway
Source: Environ Sci Technol. 2025 Jan 17;59(4):2245–56. doi: 10.1021/acs.est.4c10348 (PMC11800394; doi:10.1021/acs.est.4c10348)
Supplement: Supplementary file 1 — es4c10348_si_001.pdf [file es4c10348_si_001.pdf]

Supporting Information for

Probing the photochemical formation of hydroxyl radical from dissolved organic matter: insights into the H_2O_2-dependent pathway

Kai Cheng[1], Hang Li[1], Juliana R. Laszakovits[2], Charles M. Sharpless[3], Fernando Rosario-Ortiz[4,5], Garrett McKay[1,*]

1. Zachry Department of Civil & Environmental Engineering, Texas A&M University, College Station, Texas 77843, United States.
2. Department of Environmental Systems Science, ETH Zurich, 8092 Zurich, Switzerland.
3. Andlinger Center for Energy and the Environment, Princeton University, Princeton, NJ 08540, United States.
4. Department of Civil, Environmental and Architectural Engineering, University of Colorado, Boulder, Colorado 80309, United States.
5. Environmental Engineering Program, University of Colorado Boulder, Boulder, Colorado 80309, United States.

*Corresponding author

gmckay@tamu.edu; (979) 458-6540

Number of Pages: 29
Number of Text Sections: 8
Number of Tables: 7
Number of Figures: 13
Number of Schemes: 1

Table of Contents

Text S1 Solutions preparation 3
Text S2 Catalase activity assay 4
Text S3 Instrumental analysis 5
Text S4 Kinetic modeling of initial rates method 6
Text S5 Derivation of salicylate formation yield....................7
Text S6 Computational methods. 9
Text S7 Catalase's duality as H_2O_2 and •OH quencher. 11
Text S8 Kinetic modeling of Fe cycling through the photo-Fenton reaction 14
Table S1. List of chemicals used in this study....................16
Table S2. Characterization of whole water samples. 16
Table S3. R_{OH} measured by benzoate and the contribution by H_2O_2-dependent pathway 17
Table S4. Total iron concentration in DOM solutions 18
Table S5. Ground state ($E°$) and triplet state ($E°*$) reduction potentials for two DOM model sensitizers and their reactions leading to the formation of •OH....................18
Table S6. pK_a for two DOM model sensitizers and their radical anions 19
Table S7. Total electron density cubes with electrostatic potential (ESP) surface modeling of aqueous phase molecules....................19
Figure S1. Absolute spectral irradiance of UV lamps used in this study for all the photolysis experiments. 20
Figure S2. The stability of catalase enzyme activity over a month....................20
Figure S3. Excitation-Emission Matrix (EEM) contour plots of (A) 1 μM *p*-hydroxybenzoate and (B) 1 μM salicylate (*o*-hydroxybenzoate). 21
Figure S4. Determination of R_{OH} in 200 μM H_2O_2-sensitized system. 21
Figure S5. Determination of R_{OH} in 230 μM NO_2^--sensitized system 22
Figure S6. Control experiments demonstrating catalase's dual role as a quencher to both H_2O_2 and •OH. 22
Figure S7. Determination of the second-order rate constant between the catalase and •OH. ...23
Figure S8. Total organic carbon (TOC) and total nitrogen (TN) measurement of catalase concentration. 23
Figure S9. Catalase quenching effect to the formation •OH for secondary wastewater effluent.24
Figure S10. The concentration profile of H_2O_2 sunder UV_{365} irradiation and its stability in dark conditions. 25
Figure S11. Evaluation of the impact of photoreduction rate (k_{LMCT}) on the concentration of Fe(II) existing in the solution. 26
Figure S12. Formation of hydroxyterephthalate under UV_{365} irradiation and subsequent dark conditions.. 26
Figure S13. Control experiment using model sensitizers to evaluate the formation of •OH. 27
Scheme S1. Reaction kinetics of •OH formation (R_{OH}) and its reaction with probe molecule and scavengers. 27

Text S1 Solutions preparation.

Lab grade water (18.20 MΩ-cm resistivity) was used for all aqueous solution preparation. DOM stock solutions (~200 mg/L) were prepared by dissolving the solid isolate in lab grade water produced from a Barnstead Nanopure purification system (Thermo Scientific). NaOH (1 M) was added incrementally until a pH ~7 was reached. Following 12 hours of stirring in the dark, the 0.45 μm syringe filters (polyethersulfone, VWR) were pre-rinsed with over 50 mL of lab-grade water before use. The stock solutions were then filtered and stored in amber bottles at 4°C shielded from light. Hydrogen peroxide (H_2O_2) and sodium nitrite ($NaNO_2$) were used as model •OH sensitizers with a stock solution concentration at 20 mM and 50 mM, respectively. The concentration of H_2O_2 stock solution was verified by UV absorbance at 254 nm wavelength ($\varepsilon_{254} = 19.6\ M^{-1}cm^{-1}$). Acetophenone, *p*-benzoquinone, and 2,4,6-trimethylphenol were prepared with a concentration of 2 mM in lab grade water.

Secondary wastewater effluents were collected at the discharge of Texas A&M University Wastewater Treatment Plant in College Station, Texas. The facility employs an activated sludge treatment process followed by UV disinfection. Samples were filtered through a 1.5 μm muffled (500 °C, 4 h) glass fiber filter (Whatman GF/F) followed by a 0.45 μm MCE filter (GN-6 Metricel, Pall Corporation), stored in the dark, and kept at 4 °C prior to the experiments.

The whole water samples were collected from Town Creek in Palestine, Texas. The creek water samples were pre-treated in the same way as the wastewater effluents. Samples were filtered through a 1.5 μm muffled (500 °C, 4 h) glass fiber filter (Whatman GF/F) followed by a 0.45 μm MCE filter (GN-6 Metricel, Pall Corporation), and stored in the dark, and kept at 4 °C prior to the experiments. A portion of the creek water was treated by base modification to evaluate the role of iron (Fe) in photo-Fenton reactions. This entailed adjusting the pH of the creek water with concentrated NaOH (4 M) to pH 12. The solution was stirred for 1 hour to allow the formation of precipitates, which were then removed by vacuum filtration with a 0.45 μm MCE

filter. The filtrate was titrated back to its original pH. This process is similar to lime softening process used to remove metals and inorganic carbon.[1]

For the quantification of •OH, the probe compound reacts with •OH leading to the formation of hydroxylated products. Specifically, the monitored hydroxylated product was salicylate for benzoate and hydroxyterephthalate (hTPA) for terephthalate. The stock solutions of probe-related solutions were prepared in lab grade water with a concentration of 50 mM for benzoate, 1 mM for salicylate, and 10 mM for terephthalate, 1 mM for hydroxyterephthalate. Dissolution was facilitated by titrating the solutions with 0.1 M NaOH until pH 7 was reached.

Bovine liver catalase (Sigma Aldrich) was used as a quencher to decompose H_2O_2 produced by DOM during photolysis. A stock solution of catalase was prepared at 10 mg/mL in a total volume of 25 mL. After stirring for 2 hours, the solution was centrifuged at 4000 rpm for 20 minutes to separate the supernatant, which was then used for experiments. Catalase activity of the supernatant was evaluated by monitoring H_2O_2 absorbance decay. The details of the method can be found in Text S2. The catalase stock solution was measured with a concentration of ~10000 units/mL. The stock solution was stored at 4 °C and utilized within a month. Catalase concentrations ranging from 0 to 40 units/mL were used in the quenching experiments. Catalase activity was stable over a period of ~1 month (Figure S2).

Text S2 Catalase activity assay.

The catalase activity assay was performed to calibrate the concentration of catalase. H_2O_2 (30% w/w, Sigma Aldrich) was first diluted to 0.036% w/w to achieve an absorbance at the wavelength of 240 nm (A_{240}) between 0.550 and 0.520. Then the catalase stock solution (Text S1) was diluted by 500 times. A volume of 0.1 mL of this diluted catalase solution was mixed with 2.9 mL of the prepared 0.036% w/w H_2O_2 solution, thoroughly mixed, and placed in a cuvette for absorbance measurement. The decrease in A_{240} from 0.45 to 0.40 was monitored, with the time taken for the calculation of catalase kinetic concentration.

One unit of catalase activity is defined as the amount decomposing 1.0 μmole of H_2O_2 per minute at pH 7.0 and 25 °C. The reduction in absorbance of A_{240} from 0.45 to 0.40 corresponds to a decomposition of 3.45 μmoles of H_2O_2 in the tested solution (2.9 mL 0.036% w/w H_2O_2 and 0.1 mL catalase). Therefore, the activity-based concentration of catalase stock solution can be calculated as:

$$Units/mL\ catalase = \frac{3.45 \times dilution\ factor}{Time\ \times 0.1}$$

where the dilution factor used in the assay was 500. The time (in the unit of minute) was taken for A_{240} to decrease from 0.45 to 0.40. The number 0.1 in the equation represents the 0.1 mL volume of diluted catalase solution added to decompose the 0.036% w/w H_2O_2 solution. This protocol was adapted from the guidelines from the Enzymatic Assay of Catalase detailed on Sigma Aldrich's technical documents: https://www.sigmaaldrich.com/US/en/technical-documents/protocol/protein-biology/enzyme-activity-assays/enzymatic-assay-of-catalase.

Text S3 Instrumental analysis.

Salicylate and terephthalate were monitored using Ultra High-Performance Liquid Chromatography (UHPLC) equipped with a fluorescent detector. The specific instrument configurations for salicylate and hydroxyterephthalate are summarized in the table below. Samples should be sufficiently acidified to achieve a pH of approximately 3 before being subjected to analysis.

H_2O_2 concentration profile during DOM photolysis was monitored using the Amplex Red Assay Kit (Invitrogen). H_2O_2 in the presence of horseradish peroxidase reacts with Amplex Red at a 1:1 stoichiometry to produce resorufin, which can be determined fluorometrically using HPLC. Standard calibration was prepared daily for analysis with eight additions (0~20 μM) from diluting the H_2O_2 stock solution provided by the assay kit. A control blank (all but H_2O_2) was included to remove background interference. The Amplex Red working solution with a volume of

60 µL was mixed with 60 µL sample. After vortexing, the sample was incubated for 30 min before adding the stop reagent (Invitrogen) with a volume of 26.4 µL. UHPLC configurations for each measurement are summarized as follows:

**Parameter**	**Salicylic acid**	**hydroxyterephathalic acid**	**H_2O_2**
Column	RP-Amide	RP-Amide	C18 Ascentis
Mobile phase	70% (A) and 30% (B)	70% (A) and 30% (B)	60% (C) and 40% (B)
Flow rate	1 mL/min	1 mL/min	1 mL/min
Temperature	30 °C	30 °C	30 °C
Injection volume	50 µL	50 µL	20 µL
Fluorescent excitation wavelength	225 nm	250 nm	530 nm
Fluorescent emission wavelength	425 nm	410 nm	590 nm
Elution time	4.1 min	4.6 min	3.5 min

*C16 column (Ascentis Express 90 Å RP-Amide 15 cm × 4.6 mm, 5 µm, Supelco). Ascentis C18 selectivity column (15 cm × 4.6 mm, 5 µm, Supelco).

*Solvent (A): 10 mM orthophosphoric acid at pH 2.8 with 10% acetonitrile, Solvent (B): methanol. Solvent (C): 10 mM orthophosphoric acid at pH 7.

Text S4 Kinetic modeling of initial rates method.

The initial rates method, first employed by Zhou and Mopper,[2] was applied by varying the probe concentration to derive the formation rate of •OH. When the photosensitizer is exposed to light, •OH is photochemically produced at a rate of R_{OH} ($M\ s^{-1}$) (Scheme S1). The rate equation for probe compound (PC) loss from reaction with •OH can be written as eq. S4-1.

$$R_{PC} = -\frac{d[PC]}{dt} = k_2 \cdot [PC] \cdot [\cdot OH]_{ss} (S4-1)$$

R_{PC} represents the consumption rate ($M\ s^{-1}$) of the probe compound, $[PC]$ denotes the added concentration of probe compound (M) and k_2 corresponds to the second-order rate constant ($M^{-1}s^{-1}$) of the probe and •OH. $k_2^{BZA} = 5.9 \times 10^9 M^{-1}s^{-1}$ was used for benzoate,[3] and $k_2^{TPA} = 4.4 \times 10^9 M^{-1}s^{-1}$ was used for terephthalate.[4] $[\cdot OH]_{ss}$ represents the steady-state concentration of •OH (M) in equilibrium with the sources and sinks.

The relationship between the consumption rate of the probe compound, R_{PC} ($M\ s^{-1}$), and the formation rate of its hydroxylated product, R_P ($M\ s^{-1}$), can be expressed by eq. S4-2:

$$R_P = \frac{d[Prod]}{dt} = R_{PC} \cdot (S4-2)$$

where $[Prod]$ is the concentration of hydroxylated product monitored by UHPLC. Its cumulative concentrations were plotted against the irradiation time to calculate the R_P. The $yield$ represents the amount of hydroxylated product formed per mole of the probe compound's reaction with •OH. In this study, 0.204 was used as the yield of salicylate from benzoate. This value was calculated from internal conversion based on the relationship between the isomer ratio and yield (Text S5).[2] The validity of this number was examined in the Results section. A yield of 0.35 was used for hydroxyterephthalate.[4, 5]

[•OH]$_{ss}$ is equal to its formation rate divided by the total scavenging processes (eq. S4-3).

$$[\cdot OH]_{ss} = \frac{R_{OH}}{k_2 \cdot [PC] + \sum k_s \cdot [S]} (S4-3)$$

where $\sum k_s \cdot [S]$ represents the sum of scavenging processes, k_s is the second-order rate constant ($M^{-1}s^{-1}$) of the scavenger with •OH, and $[S]$ is the concentration (M) of the scavenger. Through connecting the three equations, it allows us to determine the R_{OH} by fitting data into eq. S4-4a, which predicts a hyperbolic dependence of the R_{OH} on the probe concentration.

$$\frac{R_P}{Yield} = R_{OH} \cdot \frac{k_2 \cdot [PC]}{k_2 \cdot [PC] + \sum k_s \cdot [S]} (S4-4a)$$

The equivalent linearized form (eq. S4-b) can be obtained by taking the reciprocal of eq. S4-4a.

$$\frac{1}{R_P/Yield} = \frac{\sum k_s \cdot [S]}{k_2 \cdot R_{OH}} \cdot \frac{1}{[PC]} + \frac{1}{R_{OH}} (S4-4b)$$

Text S5 Derivation of salicylate formation yield.

The hydroxylation reaction between benzoate and •OH results in the formation of *ortho*, *meta*, and *para*-hydroxybenzoate isomers, along with other derivatives. Consequently, the total

concentrations of these products are equal to the total concentration of •OH involved, which can be mathematically described as:

$$[\mathrm{o-BZA}]+[\mathrm{m-BZA}]+[\mathrm{p-BZA}]+[\mathrm{Others}]=[\cdot OH](S5-1)$$

The total concentration of hydroxylated benzoate isomers (ortho, meta, para) is denoted as:

$$[\mathrm{o-BZA}]+[\mathrm{m-BZA}]+[\mathrm{p-BZA}]=\sum[\mathrm{BZA}-OH\,](S5-2)$$

For *ortho*-hydroxybenzoate, the isomer ratio relative to total hydroxylated benzoate is given by:

$$\mathrm{Ratio(o)}=\frac{[\mathrm{o-BZA}]}{\sum[\mathrm{BZA-OH}\,]}(\mathrm{S5-3})$$

and yield by:

$$\mathrm{Yield(o)}=\frac{[\mathrm{o-BZA}]}{[\cdot OH]}(\mathrm{S5-4})$$

Dividing the isomer ratio by its yield derives a constant value, representing the total •OH concentration divided by the concentration of all hydroxylated benzoate isomers, as shown in equation S5-5.

$$\frac{\mathrm{Ratio(o)}}{\mathrm{Yield(o)}}=\frac{[\cdot OH]}{\sum[\mathrm{BZA-OH}\,]}(S5-5)$$

Applying the same approach to *para*-hydroxybenzoate produces equation S5-6.

$$\frac{\mathrm{Ratio(p)}}{\mathrm{Yield(p)}}=\frac{[\cdot OH]}{\sum[\mathrm{BZA-OH}\,]}(S5-6)$$

Zhou and Mopper's study provided empirical data, with isomer ratios of 36%, 34%, and 30% for (*o, m, p*)-hydroxybenzoate, and a yield for *p*-hydroxybenzoate measured as 0.17.[2] These values allowed the calculation of *ortho*-hydroxybenzoate (salicylate) yield as shown in S5-7 and S5-8.

$$\frac{Ratio(o)}{Yield(o)}=\frac{Ratio(p)}{Yield(p)}=\frac{[\cdot OH]}{\sum[BZA-OH]}(S5-7)$$

$$Yield(o)=\frac{Ratio(o)\times Yield(p)}{Ratio(p)}=\frac{36\%\times 0.17}{30\%}=0.204(S5-8)$$

It is important to note that the photoinduced *ortho*-hydroxybenzoate (salicylate) yield of 0.204 obtained from H_2O_2 sensitized system may not be indiscriminately extended to other

sensitizers where non-•OH hydroxylation may be occurring. This is underscored by the study conducted by Page. et al.[6], who investigated production of hydroxybenzoate isomers for five distinct DOM isolates and observed hydroxylation from both •OH and a non-•OH species. Cheng et al.[6] also measured the distribution of hydroxybenzoate in FeS oxygenation system. Their observation revealed the change of isomer ratios, prompting contemplation on the validity of the widely used conversion factor of 5.87 (derived by Zhou and Mopper) for *p*-hydroxybenzoic acid in systems other than H_2O_2 as the sole •OH source.[2]

Text S6 Computational methods.

The one-electron reduction potential (E^0) of acetophenone and *p*-benzoquinone as well as their protonated form, and the one-electron reduction potential of H_2O_2 were computationally calculated from Gibbs free energy of the reaction (G^*_{red}) (eq. S6-1), where n and F are the number of electrons transferred and Faraday's constant, respectively.

$$E^0 = -\frac{\Delta G^*_{red(sol)}}{nF} (S6-1)$$

Molecular geometry optimization and free energy calculations were performed with density functional theory (DFT) B3LYP[7] (all compounds) and BMK[8] (selected compounds), at 6-311+G(2df,2p) basis set using Gaussian16 platform.[9] Aqueous phase molecular geometry optimization and free energy of solvation (G^0_{solv}) were calculated with polarizable continuum model (PCM).[10] For H^+ and OH^-, reliable G^0_{solv} of cluster-based model from literatures[11-13] was used to avoid systematic errors caused by the uncertainty of absolute solvation enthalpy.

Aqueous phase free energy G^*_{sol} of the ground state molecules and the radicals in the half reactions is the sum of gas phase free energy G^0_{gas}, free energy of solvation G^0_{solv} and concentration-induced free energy shift $\Delta G^{1atm\rightarrow 1M} = 1.89\ kcal/mol$ (eq. S6-2). Gas phase free energy is the sum of electronic energy E_e and thermal free energy ΔG^0_{therm} corrected zero-point

energy ZPE in gas phase (eq. S6-3). Free energy of solvation is calculated by subtracting G_{gas}^0 to aqueous phase free energy G_{aq}^0, which was geometry-optimized and calculated with PCM (eq. S6-4).

Reduction half reactions of neutral and protonated acetophenone, *p*-benzoquinone, and H_2O_2.

$$Acetophenone\ +\ e^{-} \rightarrow Acetophenone^{\cdot -}$$

$$p-benzoquinone\ +\ e^{-} \rightarrow p-benzoquinone^{\cdot -}$$

$$Acetophenone + H^{+} +\ e^{-} \rightarrow AcetophenoneH^{\cdot}$$

$$p-benzoquinone + H^{+} +\ e^{-} \rightarrow p-benzoquinoneH^{\cdot}$$

$$H_2O_2 +\ e^{-} \rightarrow\ \cdot OH + OH^{-}$$

The free energy of reduction $\Delta G_{red(sol)}^*$ is thus calculated by subtracting G_{sol}^* of electron and the ground state molecule from G_{sol}^* of its corresponding radical (eq. S6-5 as an example of *p*-benzoquinone). E_{pH7} is equal to E^0 adjusted with H^+ or OH^- concentration of 10^{-7} M if such species present (eq. S6-6).

$$G_{sol}^* = G_{gas}^0 + \Delta G^{1atm \rightarrow 1M} + G_{solv}^0 (S6-2)$$

$$G_{gas}^0 = E_e + ZPE + \Delta G_{therm}^0 (S6-3)$$

$$\Delta G_{solv}^0 = G_{aq}^0 - G_{gas}^0 (S6-4)$$

$$\Delta G_{red(sol)}^*(pBQ) = G_{(sol)}^*(pBQ^{\cdot -}) - G_{(sol)}^*(pBQ) - G_{(sol)}^*(e^{-}) (S6-5)$$

$$E_{pH7} = E^0_{\frac{pBQ}{pBQ^{\cdot -}}} + \frac{RT}{F}\ln(Q) (S6-6)$$

The negative *log* of acid dissociation constant (pK_a) of protonated acetophenone, single-protonated *p*-benzoquinone and their radical anions was determined with their free energy of protonation ($\Delta G_{a(sol)}^*$) (eq. S6-7). The geometry optimization, gas-phase free energy calculation and solvation energy calculation of species were calculated with B3LYP/6,311+G(2df,2p) level of theory and PCM solvation model at Gaussian 16 platform. The free energy of species in

aqueous state ($G^*_{(sol)}$) was calculated with eq. S6-2, S6-3 and S6-4, which leads to the free energy of protonation ($\Delta G^*_{a(sol)}$) (eq. S6-8). $G^*_{(sol)}$ for H^+ and H_2O are cluster model data from literatures.[5–7]

$$AH^+ + H_2O \rightarrow A + H^+$$

$$AcetophenoneH^+ \rightarrow Acetophenone + H_3O^+$$

$$p - benzoquinoneH^+ \rightarrow p - benzoquinone + H^+$$

$$AH^{\cdot} \rightarrow A^{\cdot -} + H^+$$

$$AcetophenoneH^{\cdot} \rightarrow Acetophenone^{\cdot -} + H^+$$

$$p - benzoquinoneH^{\cdot} \rightarrow p - benzoquinone^{\cdot -} + H^+$$

$$pK_a(AH^+) = \frac{\Delta G^*_{a(sol)}}{RTln(10)} (S6-7)$$

$$pK_a(AH^{\cdot}) = \frac{\Delta G^*_{a(sol)}}{RTln(10)} (S6-8)$$

$$\Delta G^*_{a(sol)}(AH^+) = G^*_{(sol)}(A) + G^*_{(sol)}(H^+) - \Delta G^*_{(sol)}(AH^+) (S6-9)$$

$$\Delta G^*_{a(sol)}(AH^{\cdot}) = G^*_{(sol)}(A^{\cdot -}) + G^*_{(sol)}(H^+) - \Delta G^*_{(sol)}(AH^{\cdot}) (S6-10)$$

Text S7 Catalase's duality as H_2O_2 and •OH quencher.

In this study, the relative rate of probe hydroxylation with and without the addition of catalase was compared to quantitively determine the contribution of H_2O_2 to •OH production in DOM solutions. Initially, the catalase quenching experiment was conducted in the NO_2^- system, which does not produce H_2O_2 during photolysis.[14] The addition of catalase was expected to not affect the formation rate of hydroxylated products, thereby, for salicylate yielding a $R_{SA}^{CAT}/R_{SA}^{CAT=0}$ value equal to 1. On the contrary, Figure S6A shows that using 20 μM NO_2^- as the sensitizer with 20 μM benzoate led to a 53.2% decrease in salicylate formation at 100 units/mL catalase. This suggested that catalase acts as a •OH scavenger, which decreases the $[•OH]_{ss}$ available to benzoate and reduces the formation of salicylate. As shown by eq. S7-1, to accurately

determine the formation of •OH with and without catalase ($R_{OH}^{CAT}/R_{OH}^{CAT=0}$) by monitoring formation of hydroxylated product, like salicylate ($R_{SA}^{CAT}/R_{SA}^{CAT=0}$), the total scavenging capacity of the sensitizer and probe must sufficiently surpass that of catalase so that the scavenging competition term on the right side of the equation approaches 1.

$$\frac{R_{SA}^{CAT}}{R_{SA}^{CAT=0}} = \frac{R_{OH}^{CAT}}{R_{OH}^{CAT=0}} \times \underbrace{\frac{k_s \cdot [S] + k_2 \cdot [PC]}{k_s \cdot [S] + k_2 \cdot [PC] + k_{CAT} \cdot [CAT]}}_{\text{Scavenging competition}} (S7-1)$$

Here, R_{OH}^{CAT} and $R_{OH}^{CAT=0}$ represents the formation rate of •OH with versus without the addition of catalase, k_{CAT} stands for the second order rate constant for •OH scavenging by catalase (mL $unit^{-1}s^{-1}$), and $[CAT]$ stands for the concentration ($unit\ mL^{-1}$) of catalase.

NO_2^- acts as both a source and a sink of •OH with a significant scavenging rate constant of $1.0 \times 10^{10} M^{-1}s^{-1}$.[15] Increasing NO_2^- concentration enhances its scavenging effect to potentially reduce catalase's influence. At an 11.5-fold increase to 230 μM NO_2^-, with 20 μM benzoate, $R_{SA}^{CAT}/R_{SA}^{CAT=0}$ was increased to 79.4%, which is less affected by catalase than at lower NO_2^- levels. This is consistent with expectations based on eq. S7-1. As the concentration of the probe can be more easily controlled than the sensitizer, we raised the concentration of benzoate up to 2000 μM, with a NO_2^- concentration of 230 μM. With this adjustment, the $\frac{R_{SA}^{CAT}}{R_{SA}^{CAT=0}}$ was improved to 98% with the addition of 50 units/mL catalase, indicating minimal catalase influence on $[•OH]_{ss}$. Therefore, elevating the probe concentration to outcompete •OH scavenging of the catalase is a viable approach to quantify the H_2O_2-dependent pathway in our experimental framework.

SRHA (20 mg/L) was subsequently selected as the sensitizer to assess the contribution of H_2O_2 to the formation of •OH. Benzoate concentrations were increased progressively from 20 to 5000 μM to identify the optimal level for minimizing catalase's scavenging impact. Figure S6B shows that at probe concentrations above 500 μM, the $R_{SA}^{CAT}/R_{SA}^{CAT=0}$ values began to stabilize, indicating that benzoate at these levels predominates •OH scavenging by outcompeting the

catalase. Nevertheless, results obtained at low benzoate concentrations might be distorted by catalase interference. Utilizing eq. S4-4b, the background scavenging ($k_s \cdot [S] + k_{CAT} \cdot [CAT]$) was calculated in SRHA-sensitized solutions with catalase added from 0-100 units mL^{-1}. Figure S7 illustrates a linear relationship between increased catalase concentration in SRHA solution and the total scavenging capacity. Regression analysis estimated the •OH scavenging rate constant by catalase as 2856 ± 156 mL unit^{-1} s^{-1}. When catalase concentration is maintained below 50 units mL^{-1}, its scavenging contribution was calculated as $1.5 \times 10^5\ s^{-1}$, while benzoate at 2000 µM achieved a scavenging of $1.1 \times 10^7 s^{-1}$. The resulting •OH scavenging rate of catalase was 2 orders of magnitude lower than that from benzoate. This quantitative evidence supports using 2000 µM benzoate with catalase concentration no more than 50 unis mL^{-1} in experiments with DOM isolates to maintain adequate probe scavenging. Experiments (Figure S7) also derived a catalase •OH scavenging rate constant as $2900 \pm 200\ mL\ unit^{-1}s^{-1}$, corresponding to a scavenging rate of ~$1.45 \times 10^5\ s^{-1}$ for 50 units/mL catalase. Using an •OH reaction rate constant with organic carbon of $1.9 \times 10^4\ L\ mg_C{}^{-1}s^{-1}$, the addition of 50 units/mL catalase contributed a TOC of 8.95 ± 0.24 mg/L (Figure S8). This amount of TOC resulted in a calculated •OH scavenging rate of $1.70 \times 10^5\ s^{-1}$. These values show strong agreement and quantitively support the use of 2 mM benzoate with catalase concentration less than 50 units/mL to minimize catalase scavenging of •OH. It is important to note that catalase should be used shortly after preparation. If catalase is stored for an extended period and experiences activity loss, its effective kinetic concentration will decrease. As a result, a larger amount of catalase would need to be added to achieve the same level of activity as freshly prepared catalase. This increased catalase addition would, in turn, introduce a higher total organic carbon (TOC) concentration to the solution. Further details about the fitting model for catalase quenching used in this study can be found in the Supplementary Information of Page et al.'s publication.[6]

Text S8 Kinetic modeling of Fe cycling through the photo-Fenton reaction.

To develop the kinetic modeling, the elementary reaction pathways and their corresponding rate constants are required. The concentration profiles of involved species were calculated using differential equation solver in MATLAB. The rate law equations used to establish the modeling are listed below:

**Reactions**	**Rate constants**
$Fe(III)\xrightarrow{hv}Fe(II)$	(1) $k_{LMCT} = 0.001\sim0.01s^{-1}$
$DOM\xrightarrow{hv}H_2O_2$	(2) $k_{f(H2O2)}$
$Fe(II) + H_2O_2 \rightarrow Fe(III) + {}^{\bullet}OH + OH^-$	(3) $k_{Fenton} = 1 \times 10^4 M^{-1}s^{-1}$
$Fe(II) + {}^{\bullet}OH \rightarrow Fe(III) + OH^-$	(4) $k_{oxd} = 3 \times 10^8 M^{-1}s^{-1}$

*The formation rate of H_2O_2 from each isolate was measured in this study. The rate constants used above in the table were cited from: (1) Voelker et al.[16] and Garg et al.[17]. (3) Koppenol et al.[18]. (4) Gligorovski et al.[19].

$$\frac{d[Fe(III)]}{dt} = -k_{LMCT}\cdot[Fe(III)] + k_{Fenton}\cdot[Fe(II)]\cdot[H_2O_2] + k_{oxd}\cdot[Fe(II)]\cdot[{}^{\bullet}OH] \qquad (S8-1)$$

$$\frac{d[Fe(II)]}{dt} = k_{LMCT}\cdot[Fe(III)] - k_{Fenton}\cdot[Fe(II)]\cdot[H_2O_2] - k_{oxd}\cdot[Fe(II)]\cdot[{}^{\bullet}OH] \qquad (S8-2)$$

$$\frac{d[{}^{\bullet}OH]}{dt} = k_{Fenton}\cdot[Fe(II)]\cdot[H_2O_2] - k_{oxd}\cdot[Fe(II)]\cdot[{}^{\bullet}OH] \qquad (S8-3)$$

$$\frac{d[H_2O_2]}{dt} = k_f - k_{Fenton}\cdot[Fe(II)]\cdot[H_2O_2] \qquad (S8-4)$$

The model initializes assuming that all Fe is in the form of Fe(III), with its concentration set equal to the total Fe concentration measured via ICP-MS, as detailed in Table S4. All other species are assumed to start with zero concentration. The time-weighted average concentrations of Fe(II) was also calculated using eq. S8-5.

$$\overline{C} = \frac{1}{T}\int_0^T C(t)dt \qquad (S8-5)$$

where $C(t)$ is the concentration at time t, and T is the total duration of the time. In this model, we have used a duration of one hour, aligning with the typical length of photolysis experiments conducted in our study.

Table S1 List of chemicals used in this study.

Chemical	CAS #	Purity	Supplier
Benzoic acid	65-85-0	99.5%	Sigma Aldrich
Salicylic acid	69-72-7	99.0%	Avantor
Terephthalic acid	100-21-0	98%	Sigma Aldrich
2-Hydroxyterephthalic acid	636-94-2	97%	Sigma Aldrich
Sodium nitrite	7632-00-0	97%	Sigma Aldrich
Hydrogen peroxide	7722-84-1	30% (w/w)	Sigma Aldrich
Bovine liver catalase	9001-05-2	---	Sigma Aldrich
Amplex red assay kit	A22188	---	Invitrogen
Amplex stop reagent	A33855	---	Invitrogen
Acetophenone	98-86-2	99%	Sigma Aldrich
p-Benzoquinone	106-51-4	>98%	Sigma Aldrich
2,4,6-trimethylphenol	527-60-6	97%	Sigma Aldrich
Methanol	67-56-1	100%	VWR
Acetonitrile	75-05-8	>99.95%	VWR
Hydrochloric acid 2.0 N	7647-01-0	ACS grade	VWR
Sodium hydroxide	1310-73-2	95.0-100%	VWR
Orthophosphoric acid	7664-38-2	85%	VWR
Potassium dihydrogen phosphate	7778-77-0	>99.0%	VWR
Dipotassium hydrogen phosphate	7758-11-4	98.0%	VWR
Iron standard solution	---	---	Sigma Aldrich

Table S2 Characterization of whole water samples.

	Concentration mg/L		
**Ions**	**WWE-1**	**WWE-2**	**WWE-3**
K^+	12.45	11.54	9.47
Mg^{2+}	2.70	2.00	2.65
Ca^{2+}	18.98	15.83	BDL0.00
F^-	0.24	0.23	0.21
NO_2^-	0.59	0.56	0.15
Br^-	1.50	1.48	1.48
NO_3^-	40.14	39.51	37.47
SO_4^{2-}	49.74	46.57	46.14

*WWE represents the secondary wastewater effluent. BDL represents below detection limit.

	Concentration mg/L	
**Ions**	**Creek water**	**Creek water-BM**
K^+	5.00	BDL 0.00
Mg^{2+}	4.12	BDL 0.00
Ca^{2+}	10.83	BDL 0.00
TOC	9.75	8.48
TN	0.65	0.66

*TOC represents the total organic carbon, and TN represents the total nitrogen. BDL represents below detection limit.

Table S3 R_{OH} measured by benzoate and the contribution by H_2O_2-dependent pathway

**DOM**	R_{OH} ($\times 10^{-10}$ M/s	**H_2O_2 contribution**
SRHA	2.77 ± 0.17	13.9 ± 0.3%
SRFA	2.58 ± 0.18	10.1 ± 1.1%
SRNOM	1.66 ± 0.03	12.0 ± 0.9%
MRNOM	1.43 ± 0.07	13.1 ± 2.0%
PPHA	2.69 ± 0.20	16.3 ± 1.1%
PPFA	4.13 ± 0.24	17.3 ± 0.4%
ESHA	1.92 ± 0.02	17.9 ± 0.9%
ESFA	2.78 ± 0.06	11.7 ± 0.1%
PLFA	3.36 ± 0.09	10.8 ± 3.2%
HPOA-FL	2.23 ± 0.24	12.1 ± 0.8%
HPOA-MN	2.76 ± 0.08	15.4 ± 0.7%
HPOA-SWR	1.86 ± 0.08	14.4 ± 0.7%
WWE-1	14.9 ± 0.82	<1%
WWE-2	21.2 ± 0.46	NA
WWE-3	13.9 ± 0.40	NA
Town Creek	4.52 ± 0.38	69.9± 4.8%
Town Creek-BM	3.37 ± 0.47	7.0± 0.9%

Table S4 Total iron concentration in DOM solutions

Isolate	DOM concentration in experiment mg/L	DOM carbon percentage (%)	Fe concentration (nmol/mgC)	
			This experiment	Reference
SRHA	20	54.59	47.0	43.5[20], 42.0[21]
SRFA	20	53.3	8.3	19.8[22], 12.8[21]
SRNOM	20	50.7	33.5	109.5[22]
MRNOM	20	49.98	20.5	NA
PPHA	20	56.37	78.3	87.3[20], 67.0[23]
PPFA	20	51.31	9.6	28.7[23]
ESHA	20	58.75	13.2	33.7[20], 33.3[23]
ESFA	20	51.92	1.6	6.3[23]
PLFA	20	52.47	1.8	3.0[21]
HPOA-FL	20	53.10	0.0	5.1[24]
HPOA-MN	20	51.50	202.0	295.1[24]
HPOA-SWR	20	51.80	45.0	73.4[24]
Creek Water			1970.8	NA
Creek BM			139.6	NA
	**DOM concentration**	**DOM carbon percentage (%)**	**Fe concentration (nmol/L)**	
WWE-1	NA	NA	1574.1	

Table S5 Ground state ($E°$) and triplet state ($E°*$) reduction potentials for two DOM model sensitizers (acetophenone, ACP, and *p*-benzoquinone, *p*BQ) and their reactions leading to the formation of •OH.

Half reaction	Literature	B3LYP/6-311+G(2df, 2p)	B3LYP/6-311+G(2df,2)	BMK/6-311+G(2df,2)
	$E°$ (S/S⁻) V (SHE)	$E°$ (S/S⁻) V (SHE)	E_{pH7} (S/S⁻) V (SHE)	$E°$ (S/S⁻) V (SHE)
$ACP + e^{-} \rightarrow ACP^{\bullet -}$	-1.42[25]	-1.82	-1.82	-1.96
$pBQ + e^{-} \rightarrow pBQ^{\bullet -}$	0.099[26]	-0.098	-0.098	-0.22
$ACP + H^{+} + e^{-} \rightarrow ACPH^{\bullet}$	N.A.	-0.867	-0.453	N.A.
$pBQ + H^{+} + e^{-} \rightarrow pBQH^{\bullet}$	N.A.	0.054	0.468	N.A.
$H_2O_2 + e^{-} \rightarrow OH^{\bullet} + OH^{-}$	0.39~0.46[27, 28]	0.260	-0.154	N.A.
$TMP^{\bullet +} + e^{-} \rightarrow TMP$	1.22[29, 30]	N.A.	N.A.	N.A.
	$E°*$ (^{3}S*/ S⁻) V (SHE)	$E°*$ (^{3}S*/ S⁻) V (SHE)	$E_{pH7}*$ (^{3}S*/ S⁻) V (SHE)	$E°*$ (^{3}S*/ S⁻) V (SHE)
$^{3}ACP* + e^{-} \rightarrow ACP^{\bullet -}$	1.77[30]	1.10	1.10	1.13
$^{3}pBQ* + e^{-} \rightarrow pBQ^{\bullet -}$	2.42[30]	1.91	1.91	2.27

Table S6 pK_a for two DOM model sensitizers and their radical anions

Deprotonation Reaction	B3LYP/6-311+G(2df,2p)
	pK_a
$ACPH^{+} + H_2O \rightarrow ACP + H_3O^{+}$	-14.2
$pBQH^{+} + H_2O \rightarrow pBQ + H_3O^{+}$	-19.6
$ACPH^{\cdot} + H_2O \rightarrow ACP^{\cdot -} + H_3O^{+}$	15.4
$pBQH^{\cdot} + H_2O \rightarrow pBQ^{\cdot -} + H_3O^{+}$	1.93

Table S7 Total electron density cubes with electrostatic potential (ESP) surface modeling of aqueous phase molecules

	Acetophenone	*p*-Benzoquinone	H_2O_2
Ground state	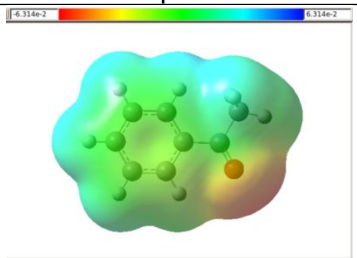	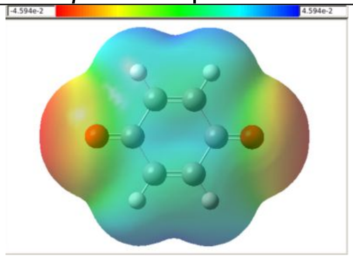	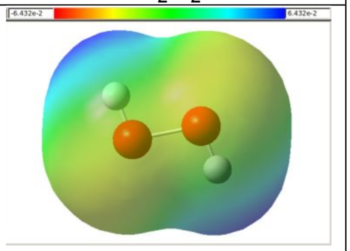
Triplet state	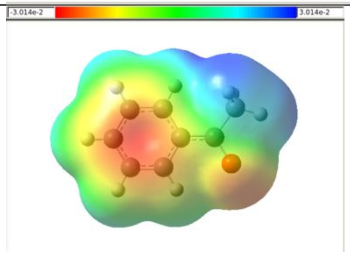	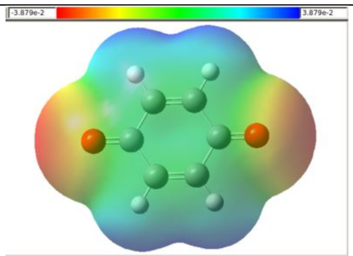	-
Radical anion	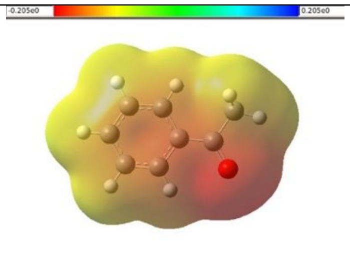	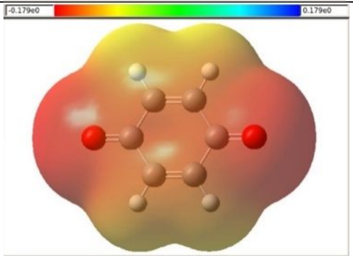	-
Radical	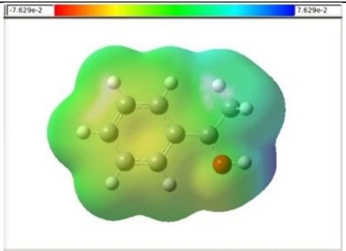	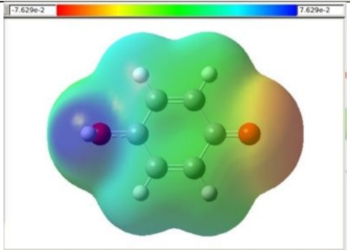	-

*The cubes show the distribution of 99.9996% total electrons in each compound, and the color contours show the ESP at the surface of the cube. Colormap end values are adapted to the min/max ESP of individual compounds. Red-yellow: negative; green: neutral; cyan-blue: positive.

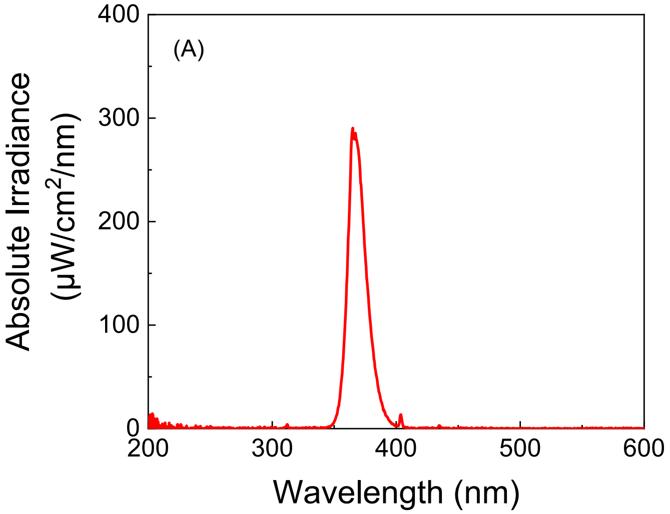

Figure S1 Absolute spectral irradiance of UV lamps used in this study for all the photolysis experiments.

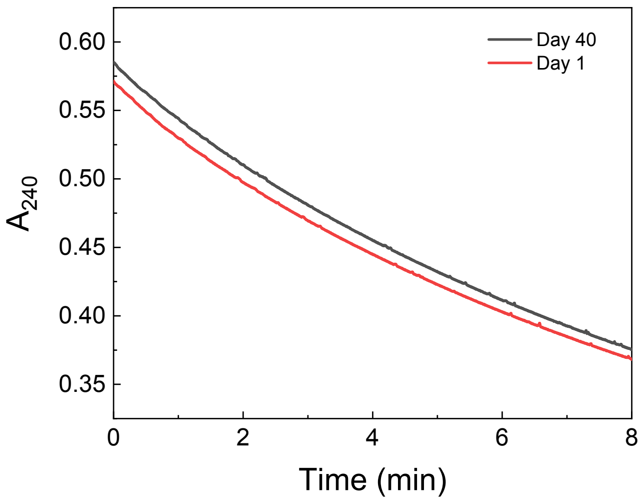

Figure S2 The stability of catalase enzyme activity over a month. The activity assays were conducted on day 1 (7094 units/mL) and day 40 (7294 units/mL) using the stock solution to assess any changes in enzyme activity. The results demonstrate consistent catalase activity over the period of usage.

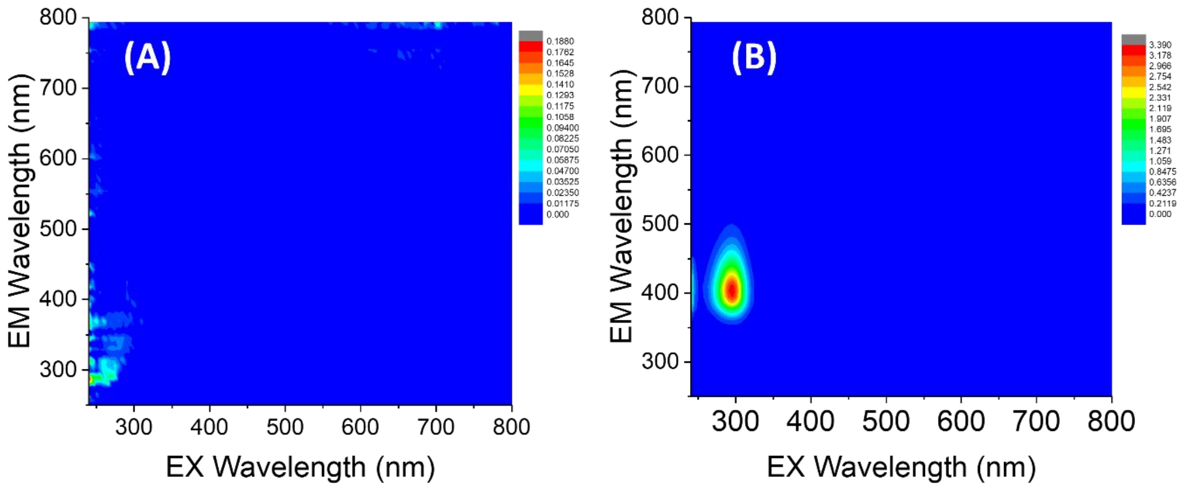

Figure S3 Excitation-Emission Matrix (EEM) contour plots of (A) 1 μM *p*-hydroxybenzoate and (B) 1 μM salicylate (*o*-hydroxybenzoate).

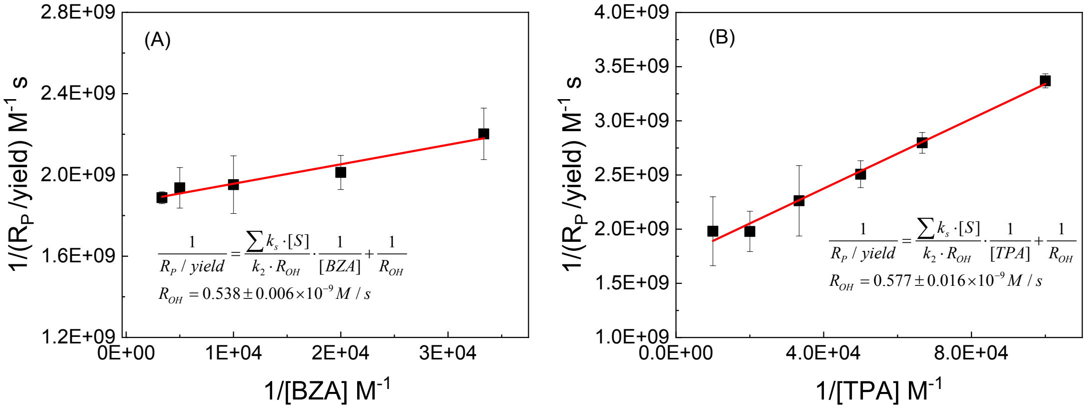

Figure S4 Determination of R_{OH} in 200 μM H_2O_2-sensitized system using equation S4-4b. (A) benzoate, used as the probe at concentrations ranging from 30 μM to 300 μM. (B) terephthalate, used as the probe at concentrations ranging from 10 μM to 100 μM (Text S4).

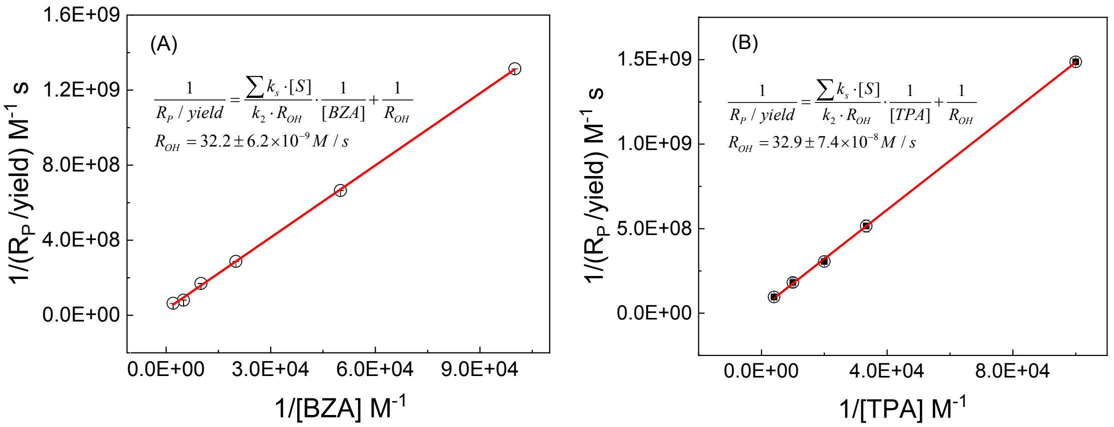

Figure S5 Determination of $\boldsymbol{R_{OH}}$ in 230 μM NO_2^--sensitized system using equation S4-4b. (A) benzoate, used as the probe at concentrations ranging from 10 μM to 500 μM. (B) terephthalate, used as the probe at concentrations ranging from 10 μM to 250 μM (Text S4).

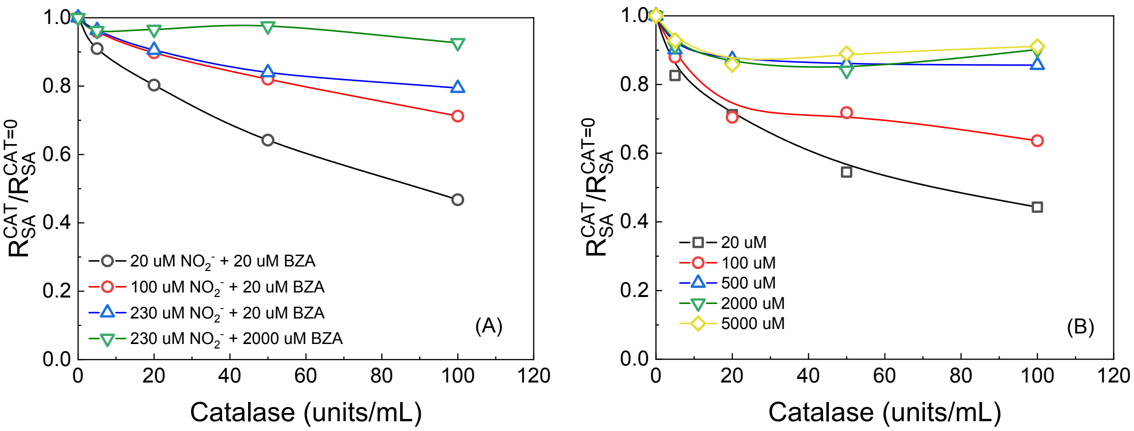

Figure S6 Control experiments demonstrating catalase's dual role as a quencher to both H_2O_2 and •OH. (A) displays the impact of catalase addition in NO_2^- system. (B) details the effect of catalase in a 20 mg/L SRHA system, where catalase is added from 0 to 100 units/mL with increasing concentrations of benzoate to outcompete the •OH radical quenching from catalase.

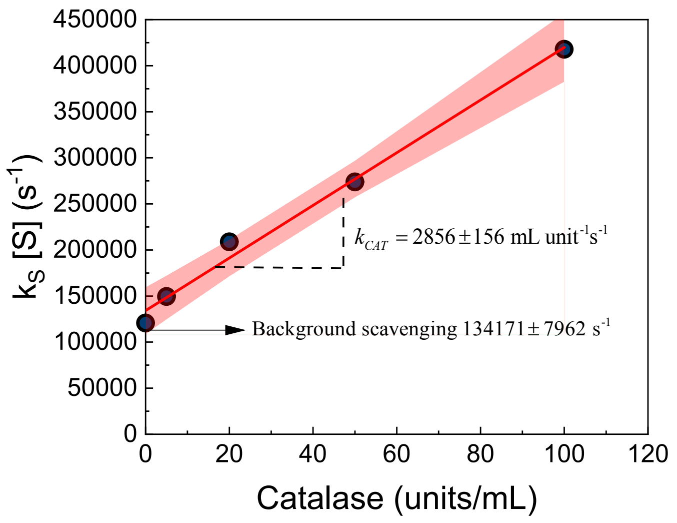

Figure S7 Determination of the second-order rate constant between the catalase and •OH. Catalase was added in an increasing concentration from 0 to 100 units/mL to 20 mg/L SRHA. Benzoate serves as the probe compound, with its concentration varying to assess the background scavenging by applying the equation S4-4b. The linear fitting with 95% confidence was performed between scavenging activity and catalase concentration is used to derive the second-order rate constant.

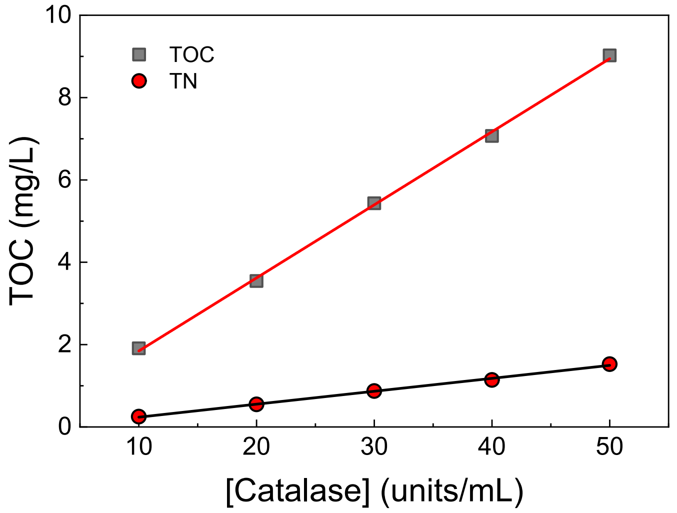

Figure S8 Total organic carbon (TOC) and total nitrogen (TN) measurement of catalase concentration.

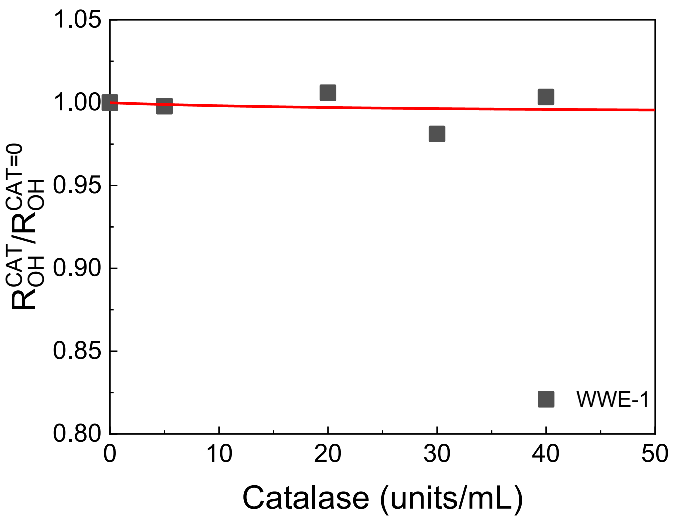

Figure S9 Catalase quenching effect to the formation •OH for secondary wastewater effluent (WWE-1).

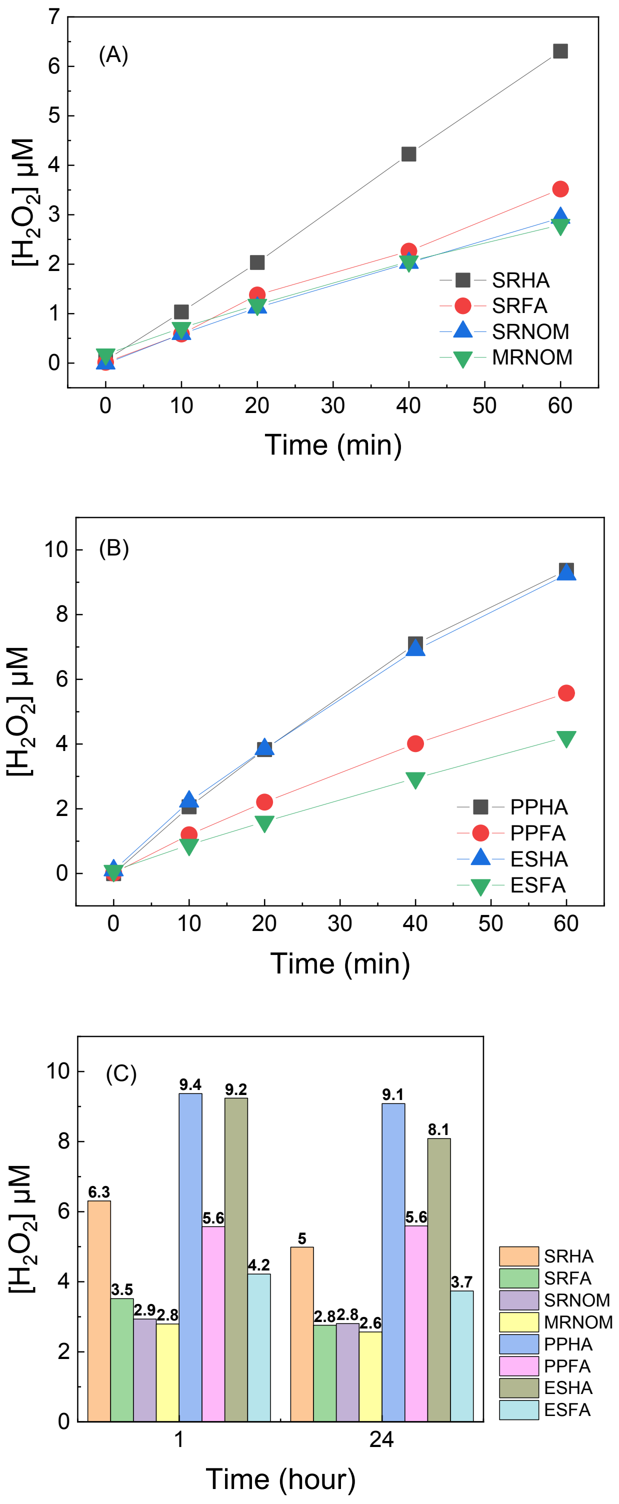

Figure S10 The concentration profile of H_2O_2 sunder UV_{365} irradiation and its stability in dark conditions. In (A) and (B) various DOM isolates were employed to determine their production rate of H_2O_2 when exposed to UV_{365} over a period of one hour; (C) H_2O_2 stability in the dark environment over 24 hours.

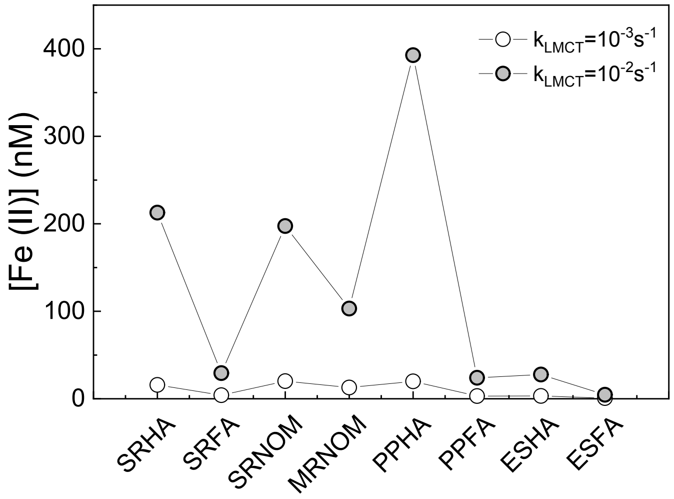

Figure S11 Evaluation of the impact of photoreduction rate ($\boldsymbol{k_{LMCT}}$) on the concentration of Fe(II) existing in the solution. Concentrations of Fe(II) were derived from modeled results using $\boldsymbol{k_{LMCT}} = \mathbf{10^{-2}{\sim}10^{-3}}\boldsymbol{s^{-1}}$ as the boundary. The data points were calculated from the time-weighted average concentration of Fe(II) derived from the model in an hour time.

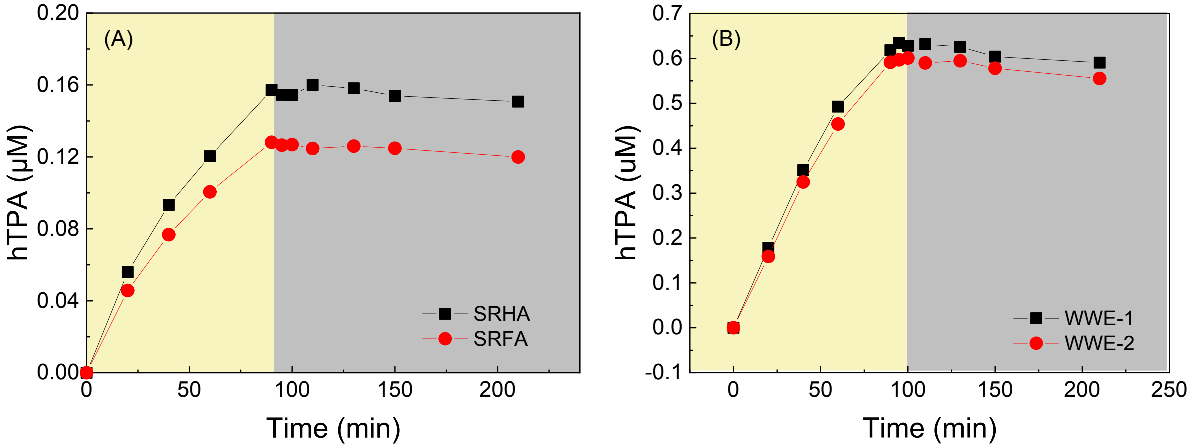

Figure S12 Formation of hydroxyterephthalate under UV_{365} irradiation and subsequent dark conditions. This figure tracks the production of hydroxyterephthalate first under UV_{365} irradiation for 90 minutes, followed by a dark period of 120 minutes to observe any continued formation of hydroxyterephthalate and its stability. (A) Shows the behavior in a 20 mg/L SRHA and SRFA solutions. (B) Details the same process in two different wastewater effluent samples.

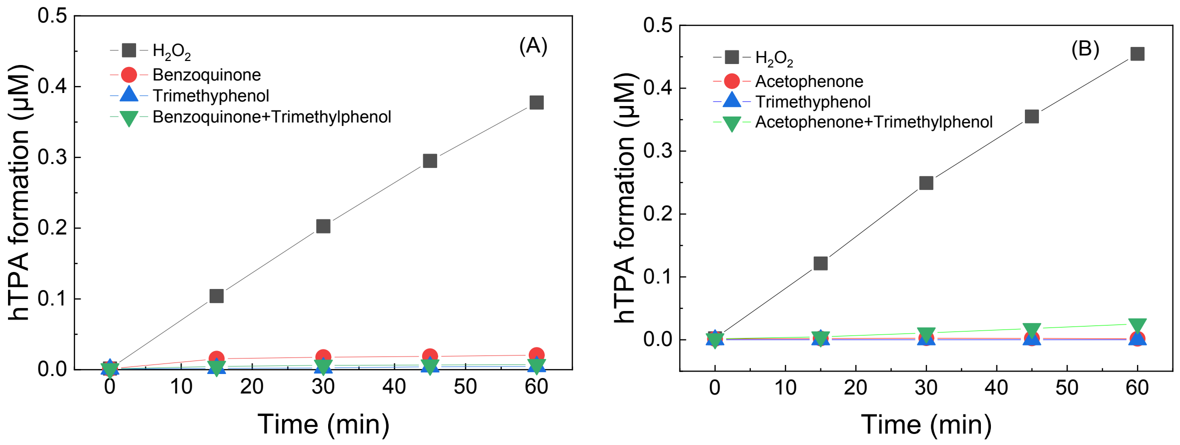

Figure S13 Control experiment using model sensitizers to evaluate the formation of •OH. These experiments employed 20 μM terephthalate as the probe to monitor the formation of hydroxyterephthalate. (A) 2,4,6-trimethylphenol showed no •OH generation under UV irradiation. In contrast, 20 μM benzoquinone alone resulted in a modest •OH generation, with a hydroxyterephthalate formation rate of $\mathbf{4.66} \times \mathbf{10^{-12}}\ \mathbf{M/s}$. (B) the mixture of acetophenone with 2,4,6-trimethylphenol resulted in a hydroxyterephthalate formation rate of $\mathbf{6.69} \times \mathbf{10^{-12}}\ \boldsymbol{M/s}$.

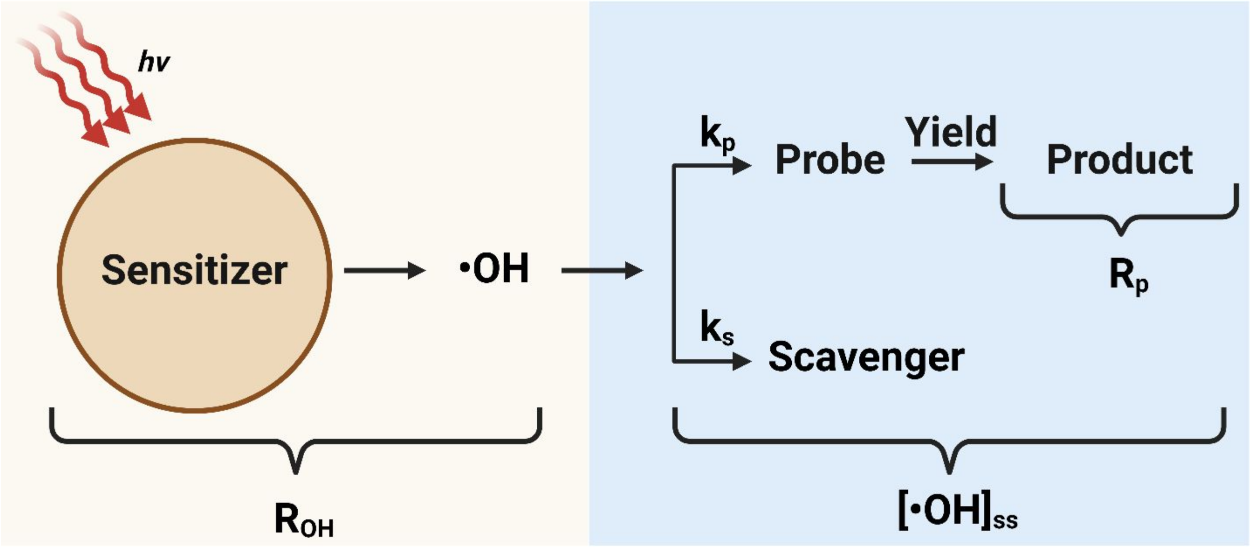

Scheme S1 Reaction kinetics of •OH formation ($\boldsymbol{R_{OH}}$) and its reaction with probe molecule and scavengers. The concentration of •OH reaches steady state in the presence of the sources and the sinks**.**

Reference

(1) Peller, J. R.; Mezyk, S. P.; McKay, G.; Watson, E. Hydroxyl Radical Probes for the Comparison of Secondary Treated Wastewaters. In *Water Reclamation and Sustainability*, Elsevier, 2014; pp 247-263.

(2) Zhou, X. L.; Mopper, K. Determination of Photochemically Produced Hydroxyl Radicals in Seawater and Fresh-Water. *Mar Chem* **1990**, *30* (1-3), 71-88. DOI: Doi 10.1016/0304-4203(90)90062-H.

(3) Buxton, G. V.; Greenstock, C. L.; Helman, W. P.; Ross, A. B. Critical-Review of Rate Constants for Reactions of Hydrated Electrons, Hydrogen-Atoms and Hydroxyl Radicals (.Oh/.O-) in Aqueous-Solution. *J Phys Chem Ref Data* **1988**, *17* (2), 513-886. DOI: Doi 10.1063/1.555805.

(4) Page, S. E.; Arnold, W. A.; McNeill, K. Terephthalate as a probe for photochemically generated hydroxyl radical. *J Environ Monitor* **2010**, *12* (9), 1658-1665. DOI: 10.1039/c0em00160k.

(5) Gonzalez, D. H.; Kuang, X. B. M.; Scott, J. A.; Rocha, G. O.; Paulson, S. E. Terephthalate Probe for Hydroxyl Radicals: Yield of 2-Hydroxyterephthalic Acid and Transition Metal Interference. *Anal Lett* **2018**, *51* (15), 2488-2497. DOI: 10.1080/00032719.2018.1431246.

(6) Page, S. E.; Arnold, W. A.; McNeill, K. Assessing the Contribution of Free Hydroxyl Radical in Organic Matter-Sensitized Photohydroxylation Reactions. *Environ Sci Technol* **2011**, *45* (7), 2818-2825. DOI: 10.1021/es2000694.

(7) Becke, A. D. Density‐functional thermochemistry. I. The effect of the exchange‐only gradient correction. *The Journal of chemical physics* **1992**, *96* (3), 2155-2160.

(8) Boese, A. D.; Martin, J. M. Development of density functionals for thermochemical kinetics. *The Journal of chemical physics* **2004**, *121* (8), 3405-3416.

(9) *Gaussian 16 Rev. C.01*; Wallingford, CT, 2016. (accessed.

(10) Miertuš, S.; Scrocco, E.; Tomasi, J. Electrostatic interaction of a solute with a continuum. A direct utilizaion of AB initio molecular potentials for the prevision of solvent effects. *Chemical Physics* **1981**, *55* (1), 117-129.

(11) Camaioni, D. M.; Schwerdtfeger, C. A. Comment on "Accurate experimental values for the free energies of hydration of H+, OH-, and H3O+". *The Journal of Physical Chemistry A* **2005**, *109* (47), 10795-10797.

(12) Lipert, R. J.; Colson, S. D. Accurate ionization potentials of phenol and phenol‐(H2O) from the electric field dependence of the pump–probe photoionization threshold. *The Journal of chemical physics* **1990**, *92* (5), 3240-3241.

(13) Tissandier, M. D.; Cowen, K. A.; Feng, W. Y.; Gundlach, E.; Cohen, M. H.; Earhart, A. D.; Coe, J. V.; Tuttle, T. R. The proton's absolute aqueous enthalpy and Gibbs free energy of solvation from cluster-ion solvation data. *The Journal of Physical Chemistry A* **1998**, *102* (40), 7787-7794.

(14) Cheng, D.; Neumann, A.; Yuan, S. H.; Liao, W. J.; Qian, A. Oxidative Degradation of Organic Contaminants by FeS in the Presence of O2. *Environ Sci Technol* **2020**, *54* (7), 4091-4101. DOI: 10.1021/acs.est.9b07012.

(15) Mack, J.; Bolton, J. R. Photochemistry of nitrite and nitrate in aqueous solution: a review. *J Photoch Photobio A* **1999**, *128* (1-3), 1-13. DOI: Doi 10.1016/S1010-6030(99)00155-0.

(16) Voelker, B. M.; Morel, F. M. M.; Sulzberger, B. Iron redox cycling in surface waters: Effects of humic substances and light. *Environ Sci Technol* **1997**, *31* (4), 1004-1011. DOI: DOI 10.1021/es9604018.

(17) Garg, S.; Jiang, C.; Waite, T. D. Mechanistic insights into iron redox transformations in the presence of natural organic matter: Impact of pH and light. *Geochim Cosmochim Ac* **2015**, *165*, 14-34. DOI: 10.1016/j.gca.2015.05.010.
(18) Koppenol, W. H. Ferryl for real. The Fenton reaction near neutral pH. *Dalton T* **2022**, *51* (45), 17496-17502. DOI: 10.1039/d2dt03168j.
(19) Gligorovski, S.; Strekowski, R.; Barbati, S.; Vione, D. Environmental Implications of Hydroxyl Radicals (center dot OH). *Chemical Reviews* **2015**, *115* (24), 13051-13092. DOI: 10.1021/cr500310b.
(20) Aeschbacher, M.; Vergari, D.; Schwarzenbach, R. P.; Sander, M. Electrochemical Analysis of Proton and Electron Transfer Equilibria of the Reducible Moieties in Humic Acids. *Environ Sci Technol* **2011**, *45* (19), 8385-8394. DOI: 10.1021/es201981g.
(21) Hudson, J. M.; Luther, G. I. I. I.; Chin, Y. P. Assessing Iron Complexation by Dissolved Organic Matter Using Mediated Electrochemical Oxidation. *Acs Earth Space Chem* **2024**. DOI: 10.1021/acsearthspacechem.4c00131.
(22) Kuhn, K. M.; Neubauer, E.; Hofmann, T.; von der Kammer, F.; Aiken, G. R.; Maurice, P. A. Concentrations and Distributions of Metals Associated with Dissolved Organic Matter from the Suwannee River (GA, USA). *Environ Eng Sci* **2015**, *32* (1), 54-65. DOI: 10.1089/ees.2014.0298.
(23) Aeschbacher, M.; Graf, C.; Schwarzenbach, R. P.; Sander, M. Antioxidant Properties of Humic Substances. *Environ Sci Technol* **2012**, *46* (9), 4916-4925. DOI: 10.1021/es300039h.
(24) Poulin, B. A.; Ryan, J. N.; Aiken, G. R. Effects of Iron on Optical Properties of Dissolved Organic Matter. *Environ Sci Technol* **2014**, *48* (17), 10098-10106. DOI: 10.1021/es502670r.
(25) Barwise, A.; Gorman, A.; Leyland, R.; Smith, P.; Rodgers, M. A pulse radiolysis study of the quenching of aromatic carbonyl triplets by norbornadienes and quadricyclenes. The mechanism of interconversion. *Journal of the American Chemical Society* **1978**, *100* (6), 1814-1820.
(26) Ilan, Y. A.; Czapski, G.; Meisel, D. The one-electron transfer redox potentials of free radicals. I. The oxygen/superoxide system. *Biochimica et Biophysica Acta (BBA)-Bioenergetics* **1976**, *430* (2), 209-224.
(27) Koppenol, W.; Butler, J. Energetics of interconversion reactions of oxyradicals. *Advances in Free Radical Biology & Medicine* **1985**, *1* (1), 91-131.
(28) Koppenol, W. H.; Stanbury, D. M.; Bounds, P. L. Electrode potentials of partially reduced oxygen species, from dioxygen to water. *Free Radical Bio Med* **2010**, *49* (3), 317-322. DOI: 10.1016/j.freeradbiomed.2010.04.011.
(29) Canonica, S.; Hellrung, B.; Wirz, J. Oxidation of phenols by triplet aromatic ketones in aqueous solution. *J Phys Chem A* **2000**, *104* (6), 1226-1232. DOI: DOI 10.1021/jp9930550.
(30) McNeill, K.; Canonica, S. Triplet state dissolved organic matter in aquatic photochemistry: reaction mechanisms, substrate scope, and photophysical properties. *Environ Sci-Proc Imp* **2016**, *18* (11), 1381-1399. DOI: 10.1039/c6em00408c.
